# A Thorough Understanding of Methylrhodium(III)–Porphyrin Photophysics: A DFT/TDDFT Study

**DOI:** 10.3390/molecules30193855

**Published:** 2025-09-23

**Authors:** Piotr Lodowski, Maria Jaworska

**Affiliations:** Institute of Chemistry, University of Silesia in Katowice, Szkolna 9, 40-006 Katowice, Poland; maria.jaworska@us.edu.pl

**Keywords:** photophysics, photodissociation, photohomolysis, intersystem crossing, ISC, internal conversion, IC, methylrhodium(III)–porphyrin, rhodium(II)–porphyrin, DFT, TDDFT

## Abstract

Rhodium–porphyrin complexes are characterised by their ability to activate C-H and C-C bonds and, therefore, find applications in synthesis and catalysis. Axial rhodoporphyrin ligands are susceptible to photodissociation under the influence of light. DFT and TDDFT calculations were performed to investigate the mechanism of photodissociation of the methyl ligand from the methylrhodium(III)–porphyrin complex (MeRhPor). Various photolysis pathways of the rhodium–methyl bond were investigated, including photolysis from states in the Q and Soret bands. Photolysis from triplet states was also considered. Based on the calculations, the most probable mechanism for photodissociation of the methyl ligand was proposed. The methyl-rhodium binding energy in the methylrhodium(III)–porphyrin complex and the energy of formation of the rhodium–porphyrin radical dimer formed by methyl dissociation were also calculated.

## 1. Introduction

Metalloporphyrins are complexes of porphyrins with transition metal ions and primarily perform important biological functions in nature, including oxygen transport using haem proteins, photosynthesis involving chlorophylls, and oxidation reactions with cytochromes P450, and are involved in numerous redox processes [1,2,3,4,5,6,7,8,9,10,11]. Many synthetic metalloporphyrins with various metal ions have been synthesised to develop artificial molecular receptors, enzyme mimics, DNA cleavers, photodynamic therapeutic agents, chiral catalysts, new materials, etc. [12,13,14,15,16,17,18,19,20,21,22]. Rhodium–porphyrins have been extensively studied over the last few decades, mainly in aspects of selective catalytic activation of C–H and C–C bonds [23,24,25,26,27].

Considering the wide range of applications of metalloporphyrins, the photophysics and photochemistry of rhodium–porphyrins play a key role in the research and application of these complexes [28,29,30,31,32,33]. Rhodium–porphyrins display strong luminescent properties due to the large aromatic network of the porphyrin ring. Alkyl substituents on the porphyrin, e.g., in OEP (octaethylporphyrin), have been shown to play a small role in the photophysical properties of the resulting complexes [34,35], whereas aryl substituents such as in TPP (meso-tetraphenylporphyrin) significantly increase the phosphorescence and fluorescence properties of these complexes. Rhodium–porphyrins exhibit moderate-to-strong phosphorescence and only weak fluorescence properties [36,37]. For five- or six-coordinate complexes, photodissociation of axial ligands is also possible. Interestingly, ligand photodissociation can occur via either a singlet or triplet photochemical pathway [38,39]. Specifically, the experimentally observed photodissociation processes involving the singlet or triplet state depend on the nature of the ligand. In the case of MeRhOEP (methylrhodium(III)–octaethylporphyrin), photodissociation of the methyl ligand is observed exclusively for the singlet photophysical channel [39]. Similarly, photodissociation of CO from (X)(CO)RhTPP (carbonylrhodium(III)–meso-tetraphenylporphyrin, X = I, Cl) occurs solely from the singlet state [38]. Since the carbonyl ligand is a strong π acceptor, it is postulated that the singlet channel applies to the photodissociation of the acceptor-type ligands. Conversely, for (Cl)(Py)RhTPP (pyridinerodium(III)–meso-tetraphenylporphyrin), photodissociation of pyridine occurs exclusively from the triplet state. Given that pyridine is a donor ligand, it is believed that the triplet state is responsible for the dissociation of the σ-type donor ligand [38]. In a situation where a ligand has both donor σ and acceptor π features, it is assumed that the ligand dissociation can occur through both singlet and triplet photophysical channels. This property was observed for the photodissociation of the isonitrile ligand (R-NC, 2,6-Dimethylphenyl isocyanide) in the (I)(R-NC)RhTPP complex [38].

**Figure 1:**
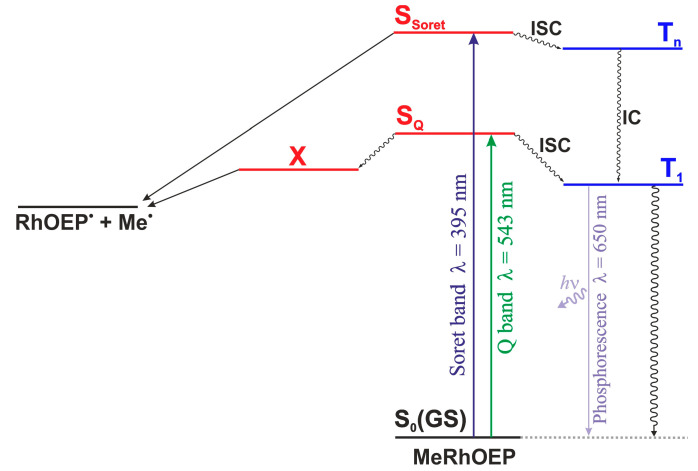
Main photochemical deactivation pathways of singlet excited states S_Q_ and S_Soret_ for the MeRhOEP complex from the perspective of experimental studies [39].

The methylrhodium(III)–octaethylporphyrin (MeRhOEP) complex, due to the simple electronic structure of the methyl axial ligand, can be considered as a model system for photophysical processes. Experimental studies of MeRhOEP photochemistry reveal the occurrence of different photophysical channels, leading to processes such as photodissociation and phosphorescence [39]. The basic photophysical paths for this complex are presented in Figure 1.

The MeRhOEP molecule absorbs light at two wavelengths, λ = 543 nm and 395 nm, which correspond to the S_Q_ and S_Soret_ singlet states, respectively. In a degassed benzene solution, there are two possible ways of deactivating excited singlet states of MeRhOEP: radiationless intersystem crossing (ISC) to the lowest triplet state and photochemical cleavage of the Rh-C_Me_ bond with involvement of only singlet states. Since, according to laser photolysis studies, there is no transition from the S_Soret_ to the S_Q_ states, the photodissociation of the Rh-C_Me_ bond occurs in two independent channels depending on the excitation wavelength region.

MeRhOEP + *hν* (λ > 410 nm) → S_Q_ → X → RhOEP^•^ + Me^•^

MeRhOEP + *hν* (λ < 410 nm) → S_Soret_ → RhOEP^•^ + Me^•^

For irradiation with light of λ > 410 nm, the temperature dependence of the photoreaction indicates that the dissociation of the Rh-C_Me_ bond in the excited singlet state requires activation energy, i.e., the intermediate X is formed thermally from the S_Q_ states, and the activation energy is estimated to be 7.4 kcal/mol [39]. Low-temperature photolysis studies revealed that photocleavage of the Rh-C_Me_ bond with irradiation light of λ < 410 nm takes place even at low temperatures, pointing to the lack of an energy barrier on the photoreaction pathway. The long-lived species X is regarded as an intermediate on the singlet path of photodissociation from the S_Q_ band; however, the structure or the electronic state of X is not known [39]. Based on the analysis of the experimental results of the quantum yield of the dissociative process and the triplet state occupation, it was concluded that the triplet state is not responsible for the photochemical reaction induced by irradiation with light of both wavelength regions λ < 410 nm and λ > 410 nm. The triplet state is deactivated through phosphorescence, which is observed at a wavelength of λ = 650 nm, and it is not involved in the process of breaking the rhodium–carbon bond.

Among the possible photophysical pathways, the channel leading to photolytic axial bond cleavage appears to be one of the most beneficial processes from the point of view of practical applications. This is related, firstly, to the relatively high energy efficiency in the case of a sufficiently high quantum yield of photohomolysis and, secondly, to the ease of control of the bond cleavage on an “on–off” basis. As long as the first aspect is important in designing photocatalytic systems, the second will find application in situations requiring the controlled release of radicals generated during photolysis. It should be emphasised here that, in general, the efficiency of a given photophysical process, including photolysis, is the result of the electronic structure of the complex but is also determined by the environment of the complex and is dependent on factors such as the nature of the solvent, the viscosity of the medium, the presence of oxygen, etc. Explaining the influence of these and other factors on photophysics is difficult without understanding the mechanistic, submolecular interaction of electronic states.

The present work describes the results of DFT and TDDFT calculations for the model structure of methylrhodium(III)–porphyrin (MeRhPor) on issues such as the equilibrium geometry of the ground state and the first excited state, the rhodium–carbon bond energy, the electronic structure and energetics of vertical excited states, and the energetics of the excited states as a function of the Rh-C_Me_ bond length, as well as the interaction of singlet and triplet states of the methylrhodium(III)–porphyrin complex.

## 2. Results

### 2.1. Geometry of MeRhPor Complex and Rh-C_Me_ Bond Dissociation Energy

**Figure 2:**
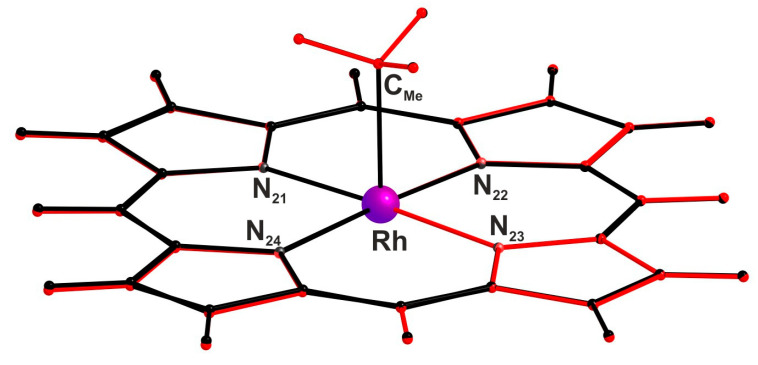
Superimposition of optimised geometries in the S_0_ and S_1_ electronic states of the MeRhPor complex: black colour—S_0_ state geometry; red colour—S_1_ state geometry. The superimposition of the geometries was performed relative to the three atoms N_24_, Rh, and N_22_.

The most relevant geometrical parameters of the coordination sphere for the methylrhodium(III)–porphyrin complex (MeRhPor) and the product of the homolytic photodissociation of the methyl radical, rhodium(II)–porphyrin (RhPor^•^), are shown in Table 1. As expected from the crystallographic data, the structure of the optimised equatorial sphere of the MeRhPor complex in the ground state S_0_, comprising the central ion and the porphyrin ligand, is almost flat. The calculated geometrical parameters of the equatorial sphere fully agree with the experimental values. The optimised axial bond length of Rh-C_Me_ is only slightly larger, by about 0.03 Å, as compared to the crystallographic data. The calculated length of this bond practically does not change for the optimised geometries of the lowest excited singlet (S_1_) and triplet (T_1_) states. Rh-N bond lengths slightly increase by ~0.01 Å and ~0.02 Å for the S_1_ and T_1_ states, respectively. At the same time, the valence and torsion angles of the coordination sphere in the excited states S_1_ and T_1_ are not significantly different compared to the geometry of the ground state (see also Table S1 in the Supplementary Materials). A superimposition of the optimised geometries in the S_0_ and S_1_ electronic states of the MeRhPor complex, presented in Figure 2, clearly shows that electronic excitation to the S_1_ state does not involve a significant change in the geometry of the complex. Therefore, it can be noted here that as a result of vertical electronic excitation S_0_ → S_1_, the geometry relaxation process in the excited S_1_ state should occur to a minimal extent. For the photolysis product, RhPor^•^, the optimised interatomic distances of Rh-N increase minimally, on average by about 0.006 Å in relation to the optimised geometry of MeRhPor in the ground state; at the same time, the values of the calculated angles indicate a completely flat geometry of the coordination sphere. In the case of the lowest singlet excited states of RhPor^•^, the geometry of the complex changes only very slightly in relation to the ground state S_0_. In the coordination sphere, the changes are essentially in the lengths of the Rh-N coordination bonds and are most probably caused by a change in the distribution of electronic density on the central ion as a result of electronic excitation.

The determined value of the Rh-C_Me_ binding energy using the PBE0 functional is 49.9 kcal/mol, and it is 8.1 kcal/mol and 4.4 kcal/mol lower than the reported experimental BDE values. Taking into account the ZPE correction and thermal corrections to the energy increases this difference by another 4 kcal/mol, giving a computationally estimated dissociation energy value of 45.8 kcal/mol. Dispersion correction considerably reduces this difference, giving values of 54.7 kcal/mol and 50.5 kcal/mol for ΔE_DE_ and ΔE_BDE_, respectively, which brings the estimated binding energy closer to the experimental values. The calculated value of 39.9 kcal/mol for the bond dissociation free energy (ΔG_BDFE_) also coincides with the experiment.

Test calculations for a widely used B3LYP hybrid functional produce a slightly larger discrepancy between the experimental data and the calculated BDE value. Including the dispersion contribution in DFT/B3LYP calculations gives practically the same ΔE_DE_, ΔE_BDE_, and ΔG_BDFE_ values as the PBE0 functional. The tested functionals of the GGA types, BP86 and PBE, give higher ΔE_DE_ bond energy values than hybrid functionals, whereas taking into account the ZPE value and thermal corrections, a bond dissociation energy (ΔE_BDE_) close to the experimental data of 54 kcal/mol is obtained. Taking dispersion corrections into account significantly increases the calculated ΔE_DE_ bond energy, and the obtained ΔE_BDE_ values are in this case close to the experimental value of 58 kcal/mol.

### 2.2. Simulated UV/VIS Spectrum of MeRhPor and Character of Excited Electronic States

**Figure 3:**
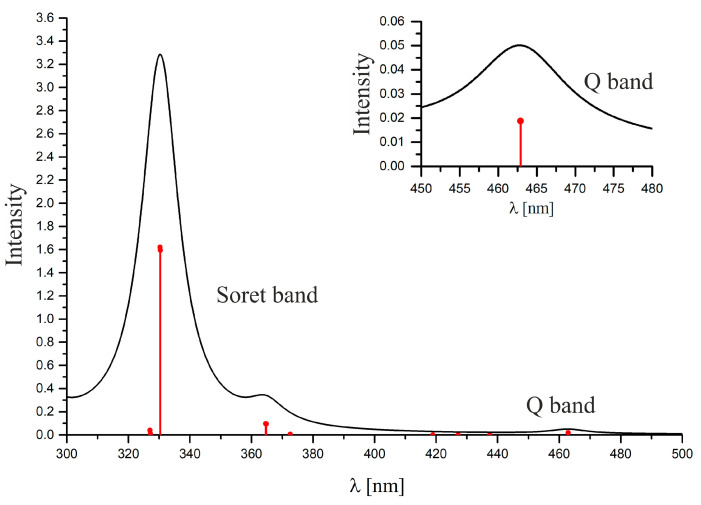
Simulated UV/VIS spectrum of the MeRhPor complex based on the TDDFT calculations. Black solid line—simulated spectral line obtained using Lorentzian broadening with a half-width of 15 nm. Vertical red lines—calculated wavelengths for vertical excitations to singlet states; the height of the line corresponds to the calculated value of the oscillator strength f.

Based on the results of calculations at the TDDFT level of theory, the absorption spectrum of the MeRhPor complex was simulated. The simulated absorption spectrum, along with the calculated excitation wavelengths and oscillator strengths, is shown in Figure 3. The simulated spectrum contains two main bands at 463 nm and 330 nm and one additional band at 364 nm. The bands at 463 nm and 330 nm can be interpreted as characteristic Q and Soret bands in the experimental spectrum. From the experiment, the position of the bands for methylrhodium(III)–octaethylporphyrin, MeRhOEP, corresponds to the following wavelengths: 543 nm, 510 nm, and 395 nm for Q(0,0), Q(1,0), and Soret(0,0), respectively [39]. Comparing the above values with each other, it is clear that the primary absorption bands in the simulated spectrum obtained from the TDDFT/PBE0 results are consistently shifted toward shorter wavelengths, including the Q band by 80 nm and the Soret band by 65 nm. Converted into energy units, this corresponds to a shift of 0.4 eV and 0.6 eV for the Q band and Soret band, respectively. Relative to the experiment, usually, such a blue shift of 50–100 nm for the main bands of the spectrum is very characteristic when hybrid functionals are used in calculations for transition metal complexes with tetrapyrrole ligands.

According to the results in Table 2, the simulated Q band is the result of electronic excitations to the first two degenerate singlet states S_1_ and S_2_, while the Soret band is a consequence of electronic transition to the degenerate states S_9_ and S_10_. The additional band in the simulated spectrum at 364 nm appears due to the presence of two states, S_7_ and S_8_, for which the calculated oscillator strength takes on a relatively higher value. It is likely that in the experimental spectrum, transitions to the S_7_–S_10_ states may form only one visible absorption band, or the actual moment of transition to the S_7_ and S_8_ states is much smaller than TDDFT calculations would suggest.

**Figure 4:**
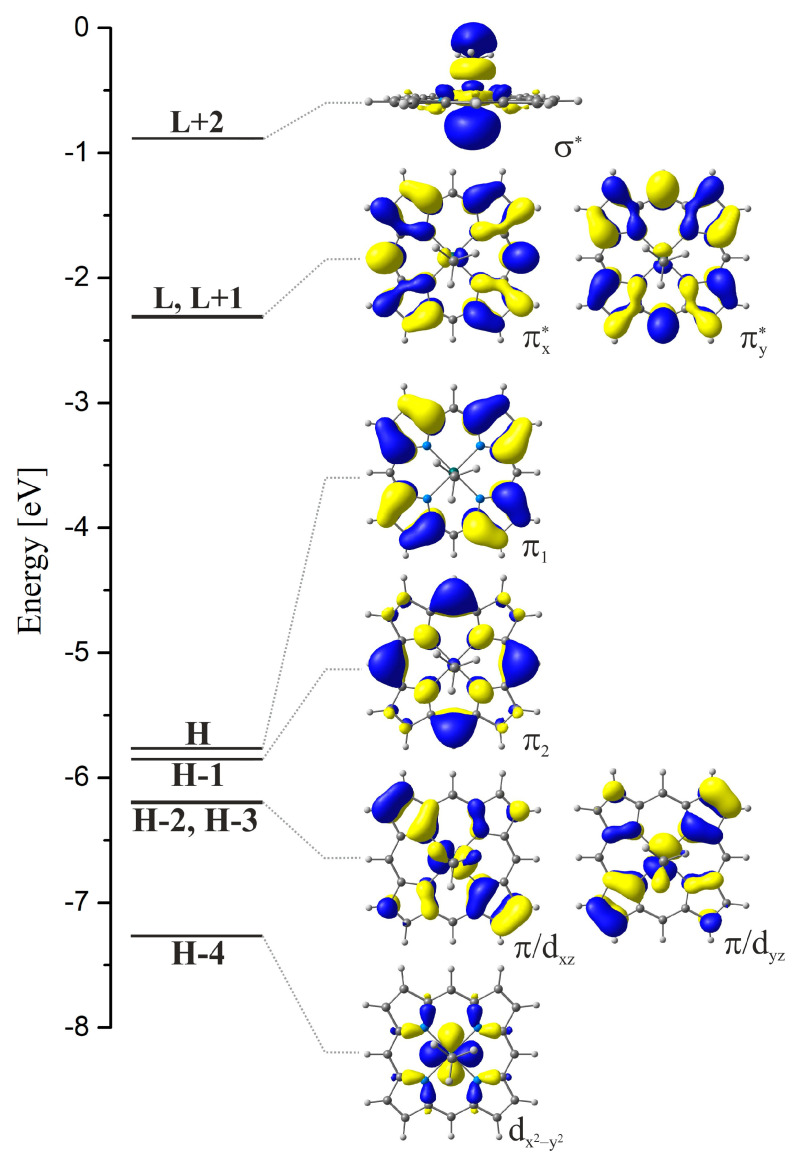
Energy diagram of frontier Kohn–Sham orbitals involved in electronic excitations for the MeRhPor complex. The shape and energetic placement of the orbitals correspond to the equilibrium geometry of the S_0_ state.

It is generally recognised that the characteristic Q and Soret absorption bands in the spectra of metalloporphyrins are the result of π → π* electronic excitations between occupied and unoccupied π orbitals localised on the porphyrin ring [44,45,46]. Also, it is assumed that the position of these bands is to some extent the result of the central ion’s interaction with the ligand. This interpretation regarding the electronic structure of singlet excited states, whose occupation as a result of photon absorption is observed in the form of the mentioned Q and Soret bands, results from the very characteristic energetic distribution of occupied and unoccupied π molecular orbitals. For the MeRhPor complex under consideration, the distribution of orbital energies within important frontier orbitals is shown in Figure 4. Due to the fact that the porphyrin ligand has a fully conjugated π-electronic system, the two highest occupied, degenerate orbitals H and H-1 and the two lowest unoccupied, also degenerate orbitals L and L+1 are π-type orbitals localised on the porphyrin ligand and have practically no admixtures of the orbitals of the central ion. Such a system of four π orbitals is commonly called Gouterman’s four-orbital model and is used to explain the absorption spectra of porphyrins. Among the frontier orbitals presented in Figure 4, the contribution of rhodium d orbitals is evident for H-4, H-3, H-2, and L+2. Orbital H-4 is a doubly occupied d_x2-y2_ orbital, and H-3 and H-2 are a combination of the d_xz_ or d_yz_ orbital with the π orbital of the porphyrin ligand, while L+2 is an unoccupied σ* orbital, which contains the d_z2_ orbital.

According to the TDDFT results given in Table 2, the first two degenerate singlet states, S_1_ and S_2_, correspond to an excitation energy of 2.68 eV and are characterised by a low value of the calculated oscillator strength. Both states are combinations of one-electron excitations between occupied H and H-1 orbitals and unoccupied L and L+1 orbitals; they are states of π → π* type. States S_9_ and S_10_ have excitations of an analogous nature; however in this case, the oscillator strengths are two orders of magnitude larger. The calculated excitation energy for these states is 3.75 eV. The remaining singlet states, presented in Table 2, are characterised by zero or medium oscillator strength and correspond to electronic excitations of varying character, but their common feature is that the Kohn–Sham orbitals involved in these transitions contain d_xz_ and d_yz_ rhodium orbitals. The S_3_–S_6_ states have a mixed π/d → π* character, whereas degenerate states S_7_ and S_8_ with an excitation energy of 3.40 eV and medium oscillator strength are of π/d → σ* type. The characterisation of singlet excited states, performed on the basis of natural transition orbital (NTO) analysis, is presented in Table S2 in the Supplementary Materials. For excited states S_1_–S_12_, the charge-transfer process in the particle–hole picture remains fully consistent with the excitation components expressed by the transitions between the frontier Kohn–Sham orbitals. Only in the case of the S_9_ and S_10_ states can a small contribution of electronic donation of the d → σ* type be observed, co-occurring with the dominant charge transfer of the π → π* character.

In Table S3 (Supplementary Materials), the triplet excited states are presented. The vertical triplet states form groups of a different character, π → π*, π/d → π*, and π/d → σ*. Below the lowest singlet states S_1_ and S_2_, four triplet states T_1_–T_4_ can be identified, appearing as two pairs of degenerate states with calculated excitation energies of 2.14 eV and 2.28 eV. The distribution of one-electron transitions characterising the electronic structure of these four states is analogous to that in the case of the singlet states S_1_ and S_2_ and S_9_ and S_10_ and corresponds to a combination of π → π* excitations. The subsequent triplet states have higher excitation energies relative to the lowest singlet states S_1_ and S_2_. Their electronic structure is more complex due to the fact that the d orbitals of rhodium are involved in electronic excitations. Triplet states T_5_–T_8_ located in the excitation energy range from 2.58 eV to 2.70 eV are of π/d → π* character. The next two states with excitation energies of 3.03 eV, T_9_ and T_10_, are of π/d → σ* type. The T_11_ and T_12_ states with an excitation energy of 3.58 eV are generally of π → π* type, and their electronic structure is similar to that of the T_1_–T_4_ states. From the point of view of MeRhPor photophysics, it is worth noting T_34_ σ→σ*, which is the non-bonding triplet state. In the paper [33], a state of this type was indicated as the state potentially responsible for the photoinduced homolysis of the Rh-C bond. However, in light of the experimental results in the work of [39], the above conclusion seems rather problematic. Based on the analysis of the decay rate of the triplet state and the quantum yield of the photodecomposition of the Rh-C bond for the aerated and degassed solution, respectively, it was concluded that the triplet state is not responsible for the photochemical reaction induced by irradiation with light of a wavelength in the regions λ < 410 nm and λ > 410 nm [39]. Thus, the mechanism of photodissociation of the axial bond emerging from the experiment indicates the singlet nature of photohomolysis. It should also be noted that the degassed benzene solution of MeRhOEP showed no ESR signal before and after photolysis. At the same time, the irradiated solution, when exposed to air, exhibits an intense ESR signal, and the ESR spectrum is in good agreement with that of the oxygen adduct of RhOEP, O_2_-RhOEP, produced from the [RhOEP]_2_ dimer [39].

### 2.3. Excited States of RhPor^•^ Complex and Dimerisation of Radical Complex

The RhPor^•^ complex is a direct product of photolytic Rh-C_Me_ bond rupture. In the ground state D_0_, the complex is a radical in which the unpaired electron occupies the d_z2_ orbital, Figure S1 in the Supplementary Materials. This location of the electronic density of the unpaired electron is the most favourable energetically and allows the formation of a two-centre Rh-Rh bond. The formation of the (RhPor)_2_ dimer can be easily confirmed computationally, as shown in Figure S2 in the Supplementary Materials. Of course, in the axial bond photolysis process, the D_0_ state is not directly accessible because the methyl group photolysis in the dissociation limit leads to an electronic state with a doubly occupied d_z2_ orbital. This fact is obvious from the point of view of the electronic excitations obtained from TDDFT calculations, the results of which are shown in Table S4 in the Supplementary Materials. First of all, the five low-lying excited states D_1_–D_5_ are of interest. The first two states are degenerate and have a π/d → d_z2_ character, which can be referred to as LMCT/LF states. The next state D_3_ is an LF-type state d → d. The next two are π → d_z2_ LMCT-type transitions. The character of these doublet states can be considered as rhodium electronic states with different electronic configurations on d orbitals. The form of the Kohn–Sham frontier orbitals involved in electronic excitations is presented in the Supplementary Materials in Figures S3 and S4. For the RhPor^•^ complex, the π Guterman’s orbital system is analogous to the π H-1, H, L, and L+1 orbital system for the MeRhPor complex (Figure S4 vs. Figure 4). The main difference between the frontier orbitals of the RhPor^•^ and MeRhPor complex is the occurrence of a single occupied d_z2_ orbital in the RhPor^•^ radical (H-7 alpha and L beta orbitals in Figure S3). In the case of the MeRhPor complex, the elongation of the Rh-C_Me_ axial bond practically does not change the character and energy position of the frontier orbitals; only in the case of σ and σ* orbitals localised on the axial bond, a significant change in orbital energy is observed, as shown in Figure S5 in the Supplementary Materials. The energy of the σ orbital increases towards the HOMO orbital, while the energy of the σ* orbital decreases, and with a significant elongation of the rhodium–carbon bond, the σ* orbital becomes the LUMO orbital. After breaking the Rh-C_Me_ bond, the d_z2_ orbital of the rhodium in the RhPor^•^ complex becomes the equivalent of the σ and σ* orbitals.

The calculated excitation energies for the D_1_–D_2_, D_3_, D_4_, and D_5_ states are, respectively, 0.42 eV, 1.17 eV, 1.76 eV, and 1.85 eV. For relaxed geometries of the D_1_, D_3_, and D_4_ states, the corresponding excitation energies are 0.40 eV, 1.17 eV, and 1.65 eV. Geometry optimisation of the D_2_ and D_5_ states at the level of the TDDFT method is impossible due to the close degeneracy of the D_1_ and D_2_ and D_4_ and D_5_ states, but it can be assumed that both the energy and geometry of these two states are the same or very close to the energy and equilibrium geometry of the D_1_ and D_4_ states, respectively. Energetically, due to the low excitation energy of about 10 kcal/mol, the D_1_ and D_2_ states can be characterised as “hot” states, whose deactivation to the ground state is a thermal process of energy dissipation due to transitions between vibronic states. From the perspective of the above calculational results, the most probable states in the photodissociation limit of the Rh-C_Me_ bond are D_1_ and D_2_ states of π/d → d_z2_ character. The relatively small difference in the energy of the states D_1_ and D_2_ and the ground state D_0_ gives rise to the conclusion that the lowest doublet excited states of the RhPor^•^ radical are quenched by internal conversion, IC, D_1_, and D_2_/D_0_, with the formation of the ground state with the configuration of the central ion (d_x2−y2_)^2^ (d_xz_,d_yz_)^4^ (d_z2_)^1^.

### 2.4. PECs as a Function of the Rh-C Bond Length

**Figure 5:**
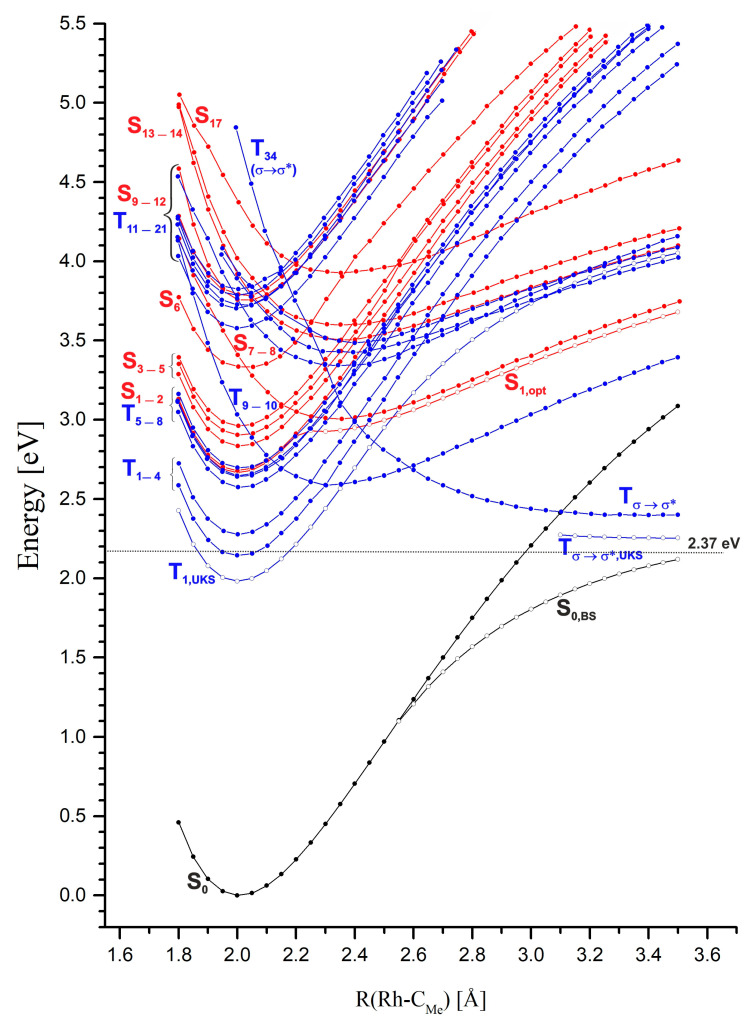
Potential energy curves (PECs) as a function of the Rh-C_Me_ distance for the ground state (S_0_) and selected vertically excited singlet and triplet states of the MeRhPor complex. Black line—PEC of S_0_ state obtained from restricted Kohn–Sham method (RKS); red lines—PECs of singlet excited states from TDDFT level of theory; blue lines—PECs of triplet excited states from TDDFT level of theory; black line with empty circles—PEC of ground state, S_0,BS_, obtained from broken-symmetry (BS) wave function; blue line with empty circles—PEC of lowest triplet state T_1,UKS_, obtained from unrestricted Kohn–Sham method (UKS); red line with empty circles—PEC of first excited singlet state S_1,opt_ obtained for optimised geometry at TDDFT level of theory.

In order to determine the photophysical mechanisms of the Rh-C_Me_ bond photodissociation process and the quenching of excited states, potential energy curves (PECs) were determined at the TDDFT level of theory as a function of the rhodium–methyl distance, shown in Figure 5 and Figure S6 in the Supplementary Materials.

**Figure 6:**
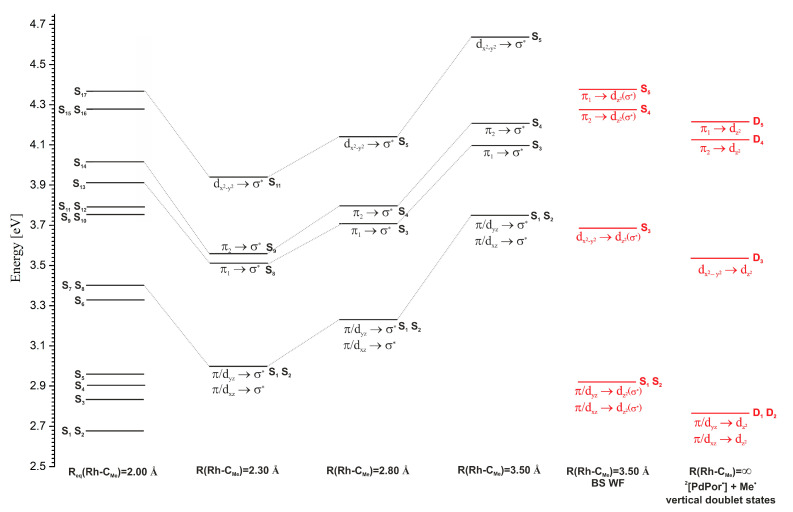
Energy diagram of selected electronic vertical states (S_7_, S_8_, S_13_, S_14_, S_17_) for different rhodium–carbon distances, R(Rh-C_Me_) = 2.00, 2.30, 2.80, and 3.50 Å, and comparison with the energies of the corresponding excited states determined by calculations with broken-symmetry wave function (BSWF) and energies of the lowest excited states of the RhPor^•^ complex (R(Rh-C_Me_) = ∞). The numbering of the electronic states for distances R(Rh-C_Me_) > 2.00 Å corresponds to their order in the given geometry.

A predominant number of PECs for excited singlet and triplet states have minima in the vicinity of the Rh-C_Me_ equilibrium distance for the S_0_ ground state, i.e., ~2.00 Å. Only in the case of a few electronic states it can be observed that their PECs have broad, shallow minima at much larger distances, usually around 2.30 Å. Among these states, the most noteworthy are the S_7_ and S_8_ states, with the π/d → σ* excitation electronic structure, which at distances greater than 2.2 Å become the lowest, excited singlet states. A similar shape of PECs is found for the S_13_, S_14_, and S_17_ states. The S_13_ and S_14_ states are of π → σ* character, and the S_17_ state is of d → σ* type. Around the 2.35 Å distance, two degenerate triplet states, T_9_ and T_10_, with the character of π/d → σ* excitation, have extensive shallow minima. In the range of distances from 2.35 Å to 2.60 Å, these are the lowest triplet states. Near the distance of 2.60 Å, the PECs of these states intersect with the curve of the T_34_ state, which is a non-bonding σ → σ* state. This curve satisfactorily converges to the calculated Rh-C_Me_ bond dissociation limit of 2.37 eV (54.7 kcal/mol). In Figure 5, a PEC fragment of the lowest triplet state, obtained at the UKS level of theory, is visible, slightly below the value of the dissociation limit. All the above-mentioned excited electronic states with a minimum on the PEC above 2.20 Å are states with electron donation to the σ* orbital of the Rh-C_Me_ bond. Thus, they are potentially dissociative states for this bond and can lead to its photolytic cleavage in the MeRhPor complex. However, the occurrence of minima on the considered PECs, especially in the case of singlet states, can be a problematic issue. First of all, it should be noted that the reference function in TDDFT calculations is a closed-shell, single-determinant wave function. Such a wave function does not correctly describe the process of homolytic dissociation of the Rh-C_Me_ bond, and in the limit of dissociation, it describes the ionic states of the products. As a result, the PESs of the excited states determined by TDDFT will not correctly describe the homolytic dissociation of the Rh-C_Me_ bond at larger rhodium–carbon distances. Calculations using a broken-symmetry wave function (BS WF) show that the PEC of the ground state S_0_ separates at a distance of 2.6 Å, and at a distance of 3.5 Å, the energy of the S_0,BS_ state is only about 0.25 eV (5.8 kcal/mol) lower than the calculated dissociation energy. For distances smaller than 2.55 Å, the BS wave function adopts the form of a closed-shell function and is equivalent to the solution of the Kohn–Sham equations in the restricted variant, giving the same total energy values as in the RKS calculations. Based on the BS function, for a distance of 3.5 Å using the TDDFT method, the excitation energy was calculated, and the character of the five lowest excited states S_1_–S_5_ was determined. The energies of the relevant states are presented in Figure 6 (R(Rh-C_Me_) = 3.50 Å, BS WF). The energy distribution of these states correlates well with the energies of the corresponding vertical states in the dissociation limit, R(Rh-C_Me_) = ∞. That is, the energy order of the states considered here coincides with the energy order of the five lowest excited states for the isolated RhPor^•^ homolysis product. On the PECs, at a rhodium–carbon distance of 3.50 Å, for five further singlet states S_1_–S_5_, the energy order of the states is different than that resulting from calculations using the BS wave function; however, above a distance of 2.20 Å, the two states of π/d → σ* character remain consistently the lowest-energy singlet states. Thus, it can be concluded that the PECs for these states as a function of rhodium–carbon bond length may not have a minimum, or the minimum is very shallow, and singlet S_1_ and S_2_ π/d → σ* states (S_7_ and S_8_ at equilibrium geometry R_eq_(Rh-C_Me_) = 2.00 Å) will be responsible for homolytic photodissociation. Preliminary calculations at the CASSCF/NEVPT2 level of theory [47,48,49,50], the results of which are presented in Figure S7 in the Supplementary Materials, reveal that the lowest singlet excited state in the Rh-C_Me_ distance range from approximately 2.40 Å to the end of the considered distance range at 4.00 Å is a state of d → σ* character. The PEC of the excited singlet state in the indicated rhodium–carbon distance range is characterised by a broad and very shallow minimum with a depth of ~0.5 kcal/mol. This confirms the above-formulated predictions that the PECs of states S_7_ and S_8_ π/d → σ* should have an almost completely dissociative character. Given the character of the next excited singlet state, S_3_ d → σ*, it can be assumed that this electronic state could also participate in photohomolysis, and in the dissociation limit, it would lead to the population of the D_3_ d → d state on the RhPor^•^ radical. Since the energy of the D_3_ state of 1.17 eV (27 kcal/mol) is relatively high, the homolysis of the Rh-C_Me_ bond with the occupation of this excited state of the RhPor^•^ radical seems to be less likely than the direct occupation of the D_1_ and D_2_ states, but ultimately, this possibility cannot be ruled out.

The potential energy curve for the optimised geometry of the first excited singlet state, S_1,opt_ in Figure 5, essentially coincides with the curve obtained for the vertical state S_1_. Understandably, the energy of this state for the optimised geometry is consistently slightly lower relative to that of the vertical state. The shape of this PEC is the result of the intersection of two singlet state curves, i.e., the lowest singlet state of π → π* character with a minimum at 2.00 Å and the higher, excited singlet state of π/d → σ* character. Although, at the TDDFT level of theory, in this case, only the one, lowest singlet state can be optimised, due to degeneracy, this curve describes the PECs of four states, S_1_ and S_2_ in the range of 1.80 Å to 2.25 Å and S_7_ and S_8_ above 2.25 Å.

### 2.5. Singlet–Triplet Interaction

For the MeRhPor complex, the occurrence of phosphorescence at wavelengths of 650 nm unambiguously indicates the presence of singlet–triplet interactions in the intersystem crossing (ISC) process. According to the Landau–Zener theory [51,52], the transition probability between the singlet and triplet states is proportional to the square of the modulus of the spin–orbital coupling integral and inversely proportional to the difference in the gradients of the intersecting PESs of the electronic states and the gradient of the change in the active coordinate over time (Appendix A). Thus, it can be stated that a large value of spin–orbital coupling, expressed by a significant value of the $H_{ST}^{SO}$ integral, transfers into an increase in the probability of the ISC S/T transition, and the probability of a singlet-to-triplet population can be estimated via the evaluation of the SOC constant (SOCC).

**Figure 7:**
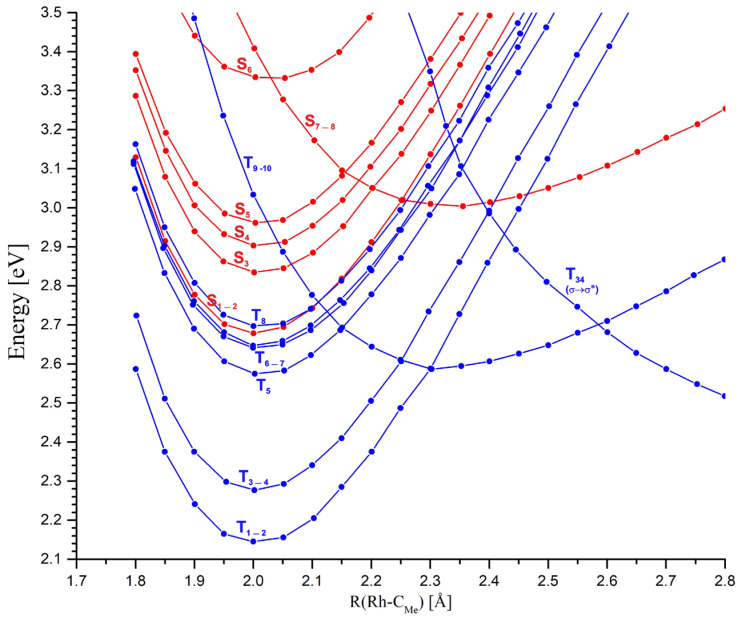
Potential energy curves (PECs) in the vicinity of the Q band as a function of Rh-C_Me_ distance for the vertically excited singlet and triplet states of the MeRhPor complex. Red lines—PECs of singlet excited states; blue lines—PECs of triplet excited states.

Radiation absorption corresponding to the Q band of the MeRhPor spectrum is associated with the occupancy of the lowest two singlet excited states S_1_ and S_2_. In the vicinity of the minimum energy of these states, four closely located T_5_–T_8_ triplet states with a similar PEC shape can be identified, as seen in Figure 7.

Calculated values of spin–orbit coupling constants (SOCCs) for selected states of the MeRhPor complex at certain Rh-C_Me_ distances are shown in Table S5 in the Supplementary Materials. The calculated SOCC value for the coupling of S_1_ and S_2_ and T_5_–T_8_ states for a distance of R(Rh-C_Me_) = 2.00 Å is in the range of 31 cm^−1^ to 35 cm^−1^. These values are not significantly large, but they indicate the possibility of weak interactions of singlet states excited in the Q band with closely located triplet states. For the two higher-energy triplet states T_9_ and T_10_, the calculated SOCC values are very small and amount to 2 cm^−1^–4 cm^−1^. In the literature [39], the possibility of a direct transition between the S_1_ and S_2_ states and lower T_1_–T_4_ states has been postulated; however, given the significant energy difference between the above-mentioned singlet and triplet states, 0.40–0.54 eV (9–12 kcal/mol), the direct interaction of these states seems unlikely. Elongation of the Rh-C_Me_ bond to 2.10 Å has practically no effect on the calculated SOCC values compared to those obtained for the equilibrium distance of 2.00 Å. Further elongation of the rhodium–carbon axial bond slightly increases the SOCC values for some triplet states of π/d → π* excitation character. However, in the case of π/d → σ* states, the SOC coefficient remains very small, ranging from 7 cm^−1^ to 5 cm^−1^ (SOCC values for S_1_/T_6_,T_7_ and S_2_/T_6_,T_7_ states at R(Rh-C_Me_) = 2.15 Å, Table S5 in the Supplementary Materials). There are two states, T_9_ and T_10_, at an equilibrium distance of 2.00 Å, whose energy rapidly decreases with an increasing rhodium–carbon bond length. Finally, at a distance of approximately 2.15 Å, the PEC of states S_1_ and S_2_ and the PEC of T_9_ and T_10_ intersect. The estimated energy barrier to the intersection point is only about 0.13 eV (~3 kcal/mol), but the direct interaction of states S_1_,S_2_ ⇝ T_9_,T_10_ via ISC is rather negligible. Firstly, this is indicated by the low SOCC value, and secondly, it must be taken into account that the PECs intersect at a significant angle. The difference in PEC gradients at the intersection point is therefore large, which, according to the Landau–Zener theory, reduces the probability of triplet states being occupied on this path. For a distance of 2.30 Å, the π → π* states S_1_ and S_2_ are close to the triplet states T_6_–T_8_ of the π/d → π* type and T_34_ with the σ → σ* character. At this distance, the calculated SOCC for the coupling between states S_1_ and S_2_ and T_6_–T_7_ is slightly higher than for the equilibrium geometry at R(Rh-C_Me_) = 2.00 Å and ranges from 45 cm^−1^ to 51 cm^−1^. In the case of the T_34_ state, the SOC coefficient of the S_1_/T_34_ and S_2_/T_34_ coupling at this Rh-C_Me_ bond length reaches a value of 69 cm^−1^. Whereas from the point of view of the estimated S/T coupling values, the probability of interaction between states S_1_ and S_2_ and triplet states may be relatively high for such an elongated axial bond, from the perspective of the photophysical mechanism, it is of rather minor importance. Elongation of the axial bond to a distance of 2.30 Å is associated with an increase in the energy of states S_1_ and S_2_ by approximately 0.46 eV (11 kcal/mol). Furthermore, at a distance of 2.25 Å, the curves of these states intersect with states S_7_ and S_8_. Since the barrier to the intersection point S_1_,S_2_/S_7_,S_8_ is ~0.32 eV (7.4 kcal/mol), it is rather unlikely that the axial bond would stretch beyond 2.25 Å along the potential energy surface of the π → π* states S_1_ and S_2_. It is more likely that IC between states S_1_ and S_2_ and S_7_ and S_8_ is the main photophysical channel for the photolysis of the rhodium–carbon bond. For the Rh-C_Me_ bond elongated to a length of 2.30 Å, the π/d → π* states S_7_ and S_8_ become the lowest excited singlet states. The interaction of these states with the π/d → π* triplet states T_5_–T_8_ can be relatively effective, considering that the calculated SOCC values range from 84 to 118 cm^−1^ depending on the specific triplet state. However, it should be considered that very flat singlet state curves intersect with triplet state curves at a relatively large angle; hence, the gradient factor at the intersection points of the curves can significantly reduce the efficiency of the ISC process for the non-radiative transition S_7_ and S_8_ ⇝ T_5_–T_8_. For a distance of 2.40 Å, the PEC of the lowest-energy singlet states, S_7_ and S_8_, intersects the curves of the π → π* states of T_3_ and T_4_, as well as the T_34_ non-bonding state. For the T_3_ and T_4_ states, the calculated SOCC value for the interaction with the S_7_ and S_8_ states is not large, ranging from 21 cm^−1^ to 34 cm^−1^, while for the T_34_ state, it is very large, equal to 533 cm^−1^. This result may suggest that the probability of occupying the triplet state is high and that a non-radiative transition to the triplet homolysis pathway would be possible during singlet photodissociation of the methyl group. However, as in the case of the other triplet states whose PECs intersect the curves of the S_7_ and S_8_ states, the S_7_,S_8_/T_34_ intersection also occurs at a large angle, which certainly increases the gradient factor and thus reduces the probability of a direct transition of the singlet states to the triplet σ → σ* state. With further elongation of the axial bond to a distance of 2.45 Å, the intersection of the singlet state curves with the PEC of T_1_ and T_2_ triplets is apparent. The calculated value of the SOCC for the coupling of these states is very small and not greater than 14 cm^−1^. Thus, a direct interaction of the singlet states with the lowest triplet states is unlikely to be possible in the photophysical path corresponding to the elongation of the rhodium–carbon bond.

**Figure 8:**
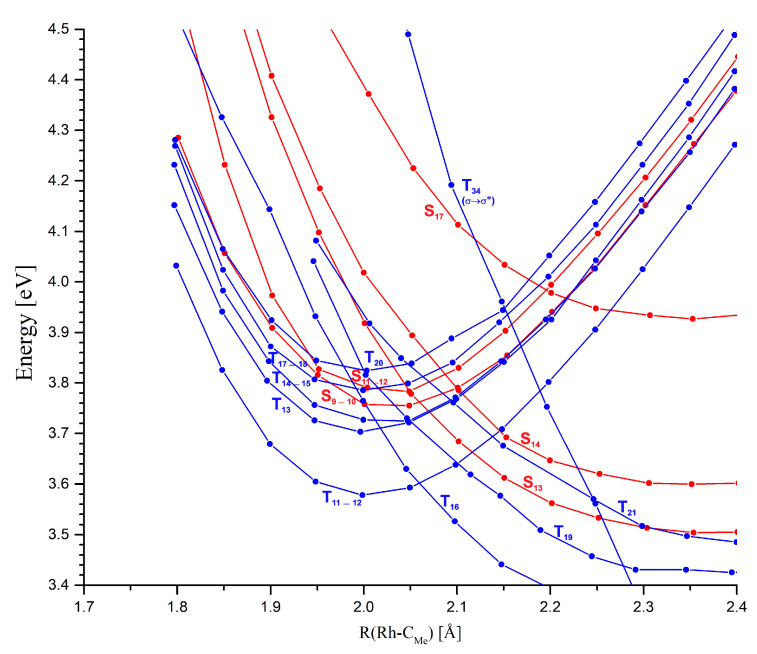
Potential energy curves (PECs) in the vicinity of the Soret band as a function of Rh-C_Me_ distance for vertically excited singlet and triplet states of the MeRhPor complex. Red lines—PECs of singlet excited states; blue lines—PECs of triplet excited states.

Excitation into the Soret band corresponds to the occupancy of mainly two singlet electronic states, S_9_ and S_10_ of the π → π* type. Near the energy minimum of these states, nine closely spaced T_13_-T_21_ triplet states appear, of which the PECs of the T_13_–T_18_ states have a curvature similar to the PECs of the singlet states, with a minimum for R(Rh-C_Me_) = ~2.00 Å, as depicted in Figure 8. In general, for states T_13_ to T_21_, the calculated SOC coefficient value varies greatly, from low to medium to relatively high. In addition, these values vary somewhat depending on the singlet state interacting with a given triplet. The calculated SOC coefficients of medium value mainly refer to the interaction with the triplet states T_16_, T1_8_, and T_19_ with π → σ*, π/d → d, and d → σ* characters, respectively, and their range is between 21 and 52 cm^−1^. The higher value is reached by the SOC coefficient for the coupling of the S_9_ and S_10_ states with the T_16_ and T_21_ states with the π → σ* character, and it is in the range of 21 cm^−1^ to 75 cm^−1^, while the highest value of the coefficient corresponds to the coupling of the S_9_ and S_10_ singlet states with the T_17_ π/d → d state, where the SOCC reaches about 200 cm^−1^. Thus, analysing the singlet–triplet interactions within the equilibrium geometry of the S_9_ and S_10_ states, it can be concluded that the probability of occupying the triplet states after excitation in the Soret band is relatively high.

Elongation of the axial bond slightly increases the SOCC for the interaction of singlet π → π* states with triplet states of type π → σ*, and at a distance of 2.15 Å, the value of the coupling coefficient is 84 cm^−1^–92 cm^−1^. Also, at the same distance, the SOCC for the interaction with triplet T_19_ of type d → σ* increases and is 77 cm^−1^ and 107 cm^−1^ for the coupling of states S_9_/T_19_ and S_10_/T_19_, respectively. As the bond length of Rh-C_Me_ increases, the energy difference between the singlet and triplet states considered above grows, and the PECs of these states move away from each other, so a virtually barrier-free, direct crossing of the potential surface of the triplet states T_16_, T_19_, and T_21_ with the surface of the states S_9_ and S_10_ occurs in the vicinity of the equilibrium geometry of the singlets. Due to the strong compaction of triplet states in the vicinity of the minimum and the occurrence of the PEC intersections in close proximity to it, qualitative estimation of the influence of the gradient factor on the probability of S ⇝ T transitions is basically impossible. At a distance of 2.15 Å, a pronounced coupling of singlet states with the non-bonding triplet state σ → σ* also appears, and the SOCC for this coupling is about 108 cm^−1^. The estimated energy barrier to the PEC intersection of S_9_ and S_10_ and T_34_ in this case is ~0.1 eV (~2.3 kcal/mol). Although the energy barrier is not significant, the PEC curves intersect at a rather large angle, which increases the gradient factor and reduces the probability of ISC between the S_9_, S_10_, and T_34_ states.

## 3. Discussion

**Figure 9:**
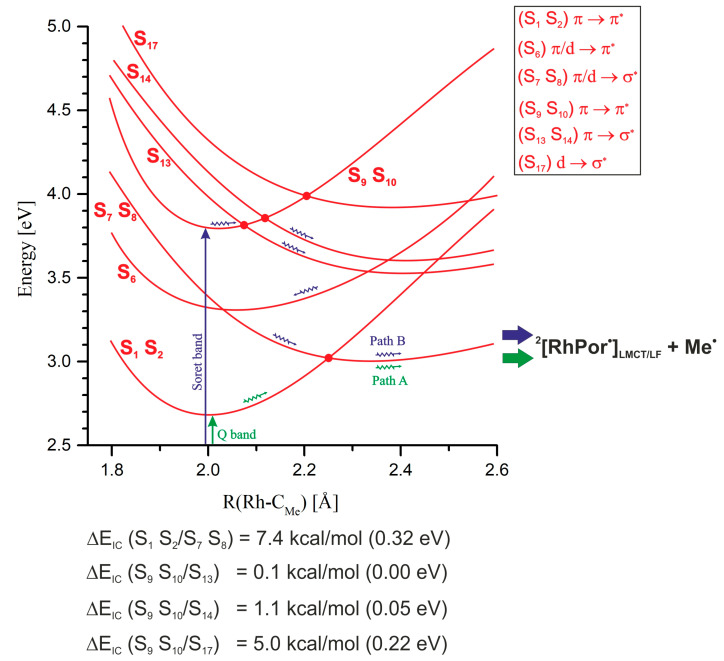
PECs of the most important excited singlet states involved in the photophysics of the MeRhPor complex and the predicted pathways of IC and photohomolysis of the Rh-C_Me_ axial bond.

Based on the obtained results, a mechanism can be proposed for singlet photophysical pathways leading to internal conversion (IC) and photodissociation of the rhodium–methyl bond, which is schematically shown in Figure 9.

Absorption in the Q band (exp. 543 nm, calc. 463 nm) excites the MeRhPor molecule to two degenerate π → π* states, S_1_ and S_2_ (generally S_Q_). After excitation, the molecular geometry practically does not relax, as the equilibrium geometries of the ground state S_0_ and the excited states S_1_ and S_2_ are not significantly different from each other. In these two lowest electronically excited singlet states, the molecule is stabilised in a relatively deep minimum on the potential energy surface. The S_1_ and S_2_ states could possibly be quenched by the S_1_ and S_2_ ⇝ S_0_ fluorescence process, but experimental studies do not indicate the occurrence of this process. Alternatively, deactivation may occur by elongating the axial bond with the methyl ligand. Elongation of the Rh-C_Me_ bond leads to internal conversion between the S_1_ and S_2_ and π/d → π* S_7_ and S_8_ states above 2.2 Å. The computationally estimated energy barrier of this IC process is 7.4 kcal/mol and correlates well with the experimental value of 7.4 ± 1.2 kcal/mol for the MeRhOEP complex photodecomposition barrier [39]. The degenerate S_7_ and S_8_ states are non-bonding or weakly bonded states, and their population will ultimately lead to radical homolysis of the Rh-C_Me_ bond. The photophysical pathway between the relaxed states S_1_ and S_2_ and the products of axial bond rupture is marked with small green arrows in Figure 9 and described as path A. As a result of photohomolysis, the final product of the decomposition is a methyl radical and a RhPor^•^ complex in the D_1_ and D_2_ π/d → d_z2_ excited doublet states, denoted as ^2^[RhPor^•^]_LMCT/LF_ in Figure 9. The quenching of the D_1_ and D_2_ states in the RhPor^•^ complex is a thermal process occurring as vibrational energy dissipation and leads to the formation of a radical complex with a singly occupied d_z2_ rhodium orbital. Further processes, such as the formation of a two-centred [RhPor]_2_ dimer complex, radical recombination, and interaction with the external environment within the solvent cage are the result of the solvent character and the dynamics of the processes depending on temperature, medium viscosity, MeRhPor concentration, etc. Experimental findings suggest the existence of an additional state mediating the homolysis of the axial bond, S_1_ → X → RhPor^•^ + Me^•^, in which the excited complex undergoes direct decomposition to radical products and in the presence of oxygen undergoes a direct radiationless deactivation to the ground-state MeRhPor [39]. In light of the computational results discussed above, the intermediate state X may be the degenerate states S_7_ and S_8_ available through IC during Rh-C_Me_ bond elongation. It is probable that the delay in the bond-breaking process in these states is not directly related to their electronic structure but to the above-mentioned dynamics of the processes accompanying photohomolysis, considering the fact that the kinetic factor (the velocity of the decomposition products) is insufficient to quickly overcome the barrier associated with the relaxation of the solvent cavity during the rupture of the bond. Therefore, the structure/electronic state X formation seen in the experiment may be associated with an increased bond-breaking time in the intermediate states S_7_ and S_8_. In the case of ultrafast reactions like photochemical bond cleavage, where strong changes in the molecular structure occur, the solvent molecules directly interact with the molecular motion of the solute. The solute’s fragments, moving apart, push into the solvent cage, and their initial motion can be significantly hindered [53,54].

Absorption in the Soret band (exp. 395 nm, calc. 330 nm) involves excitation to higher singlet excited states of π → π*, S_9_ and S_10_ (generally S_Soret_). By analogy with the S_1_ and S_2_ states, it should be thought that also in this case, after vertical excitation, the geometry of the complex in the excited state does not undergo much change in order to reach the minimum on the potential energy surface. In contrast to the situation in the S_1_ and S_2_ states, where the energy minima occur at a greater distance from the intersections with the potential energy surfaces of other states, in the case of excited states in the Soret band, the minima are closer to such intersections. With a slight elongation of the Rh-C_Me_ bond in the range of ~0.5 Å to ~1.0 Å, first of all, the PECs of the S_9_ and S_10_ states intersect with the curves of the S_13_ and S_14_ states with a π/d → σ* character. The possible intersection points are close enough to the minima so that the processes of internal conversion of S_9_,S_10_ ⇝ S_13_,S_14_ are practically barrierless. This IC occurrence starts a cascade of successive geometry relaxations and a series of subsequent internal conversions, involving a sequence of increasingly lower-energy excited states: S_9_,S_10_ ⇝ S_13_,S_14_ ⇝ S_6_ ⇝ S_7_,S_8_, as is shown by the small blue arrows in Figure 9. As a result, the S_7_ and S_8_ states are occupied, which leads directly to photolysis of the axial bond. The photophysical pathway discussed above, labelled path B in Figure 9, is virtually devoid of energy barriers. From the perspective of experimental results, it is said that the MeRhPor complex undergoes direct photochemical decomposition after excitation with a wave λ < 400 nm, which also includes the Soret band. Of course, in the context of the presented computational results, one can speak of rapid but rather not direct photodissociation. The experimental results also do not indicate the presence of intermediate stages in the case of photodissociation from S_Soret_ states. Although the final stage of photolysis of the Rh-C_Me_ bond, involving the S_7_ and S_8_ states, is common to both paths A and B, it can be assumed that the kinetic factor has a significant impact on the dynamics of the final stage of photodissociation. Due to the high energy of the S_Soret_, bond dissociation will occur with a much higher kinetic energy of decomposition fragments compared to the bond rupture from the S_Q_ states, and this applies in particular to methyl radical formation. In this case, the cleavage rate of the Rh-C_Me_ bond in states S_7_ and S_8_ may be fast enough that homolysis occurs rapidly before the geometry of the complex has time to relax to states S_1_ and S_2_ as a result of IC, and simultaneously, it could be said that there is no transition from the S_Soret_ to the S_Q_ states.

**Figure 10:**
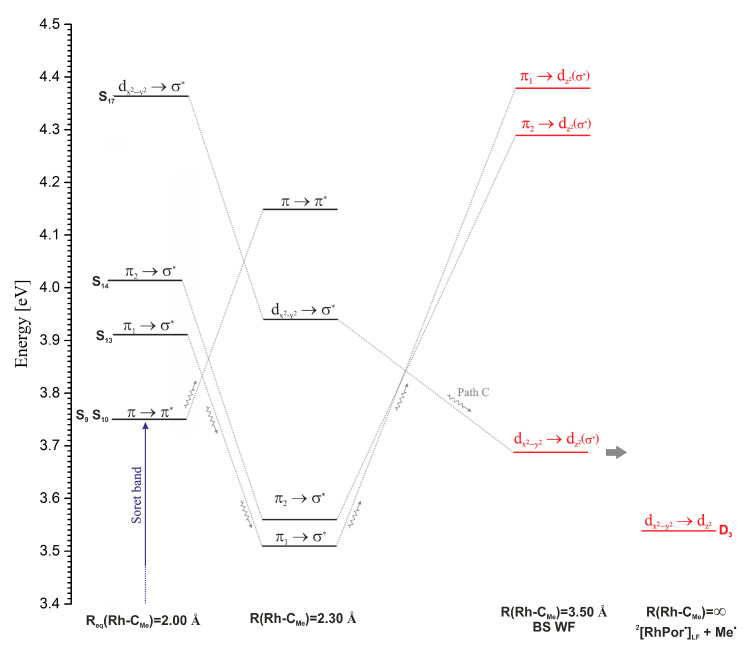
Energy diagram of singlet excited states of the MeRhPor complex involved in the photolysis of the Rh-C_Me_ bond after excitation to S_Soret_ states (S_9_ and S_10_).

However, there are some indications that direct photolysis of the Rh-C_Me_ bond may occur with involvement of the states S_13_, S_14_, and S_17_, as is shown in Figure 10. The direct path involving S_9_ and S_10_ ⇝ S_17_ internal conversion is rather unlikely because such IC occurs with a much more elongated axial bond than is the case for the S_13_ and S_14_ states and is associated with a higher energy barrier of about 5 kcal/mol. Concerning path B, the internal conversion S_9_,S_10_ ⇝ S_13_,S_14_ is more likely in the first stage of deactivation of S_Soret_ states. Ultimately, the photohomolysis of the axial bond only through states S_13_ and S_14_ would have to lead to the formation of the RhPor^•^ product in a relatively highly excited state (states D_4_ and D_5_ in Figure 6), which is very unlikely. Regarding the TDDFT method in a closed-shell version, the potential energy curves of the excited states are heading toward incorrect dissociation limits. The analysis of the characters and energetics of singlet states based on the BS wave function at large Rh-C_Me_ distances and at the dissociation limit indicates that the potential energy surface of the high-energy d → σ* state (S_17_) should intersect the surfaces of the π → σ* states (S_13_, S_14_) as the axial bond lengthens. Such intersections open an alternative path towards homolysis and lead to the formation of the RhPor^•^ final product in the excited state of D_3_ d → d_z2_. Thus, a photolysis path can be proposed in the form of the following sequence: S_9_,S_10_ ⇝ S_13_,S_14_ ⇝ S_17_ ⇝ ^2^[RhPor^•^]_LF_ + Me^•^ (Figure 10).

Although the energy of state D_3_ is relatively high, considering its character, it can be assumed that, like states D_1_ and D_2_, it is thermally quenched. In such a mechanism, partial quenching of the energy of the S_Soret_ states would take place on the photolysis product. Unfortunately, based on the results discussed here, it is not possible to demonstrate a clear preference for the occurrence of a B or C path in the photodissociation process of the axial bond. This requires further, more detailed research, both theoretically and experimentally. Although the mechanism based on path C practically excludes the possibility of the creation of an X state, which is in good agreement with the interpretation of the experimental results, the creation of a photolysis product in a higher, excited state may be debatable. On path B, thermal quenching of the excitation energy takes place mainly as a relaxation of the states with an activated Rh-C_Me_ bond, while on path C, ~30% quenching of energy should take place with the participation of the RhPor^•^ photolysis product.

**Figure 11:**
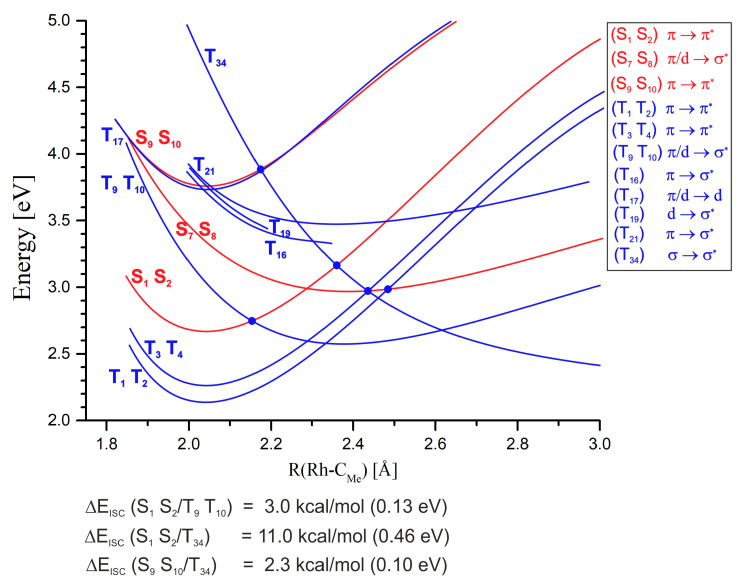
PECs of the most important excited singlet and triplet states involved in the photophysics of the ISC process for the MeRhPor complex.

According to the experimental findings, in the excitation wavelength range 410 < λ < 550 nm, the MeRhOEP molecule, after excitation, is directed to the triplet state or forms the photodecomposition product from excited singlet states. Therefore, from the perspective of the experimental findings, MeRhPor photophysics must also involve intersystem crossing (ISC) processes for the forbidden transitions between excited singlet and triplet states. The population of triplet states through the ISC channel results in the occurrence of phosphorescence due to the deactivation of the lowest π → π* triplet states. Experimentally, the phosphorescence band is observed at a wavelength of λ = 650 nm, which correlates very well with the calculated value of 653 nm determined on the basis of vertical deexcitation energy for the optimised T_1_ state geometry using the UKS formalism. Since the main singlet photophysical channel leads to the rupture of the Rh-C_Me_ bond, primarily the possibilities of S/T crossing by changing the R(Rh-C_Me_) distance should be considered. By analysing the PECs as a function of the R(Rh-C_Me_) coordinate, for the S_Q_ singlet states, two intersection points of the energy curves of electronic states S_1_ and S_2_ and T_9_, T_10_, and T_34_ can be indicated, as shown in Figure 11. To reach the first point, where the PECs of the S_Q_ π → π* states intersect with the T_9_ and T_10_ π/d → σ* states, the system has to overcome a small energy barrier of about 3 kcal/mol. However, due to the low value of the calculated SOC coefficient, ≤4 cm^−1^, and the presence of a clearly unfavourable gradient factor, such an ISC channel of the triplet state population is rather unlikely. In the case of the second intersection point, where the PECs of the S_Q_ states intersect the triplet energy curve of the T_34_ state of σ → σ* character, the calculated SOCC value is 69 cm^−1^; nevertheless, the importance of this ISC channel for occupying triplet states is rather minor. As emphasised in Section 2, this point occurs at a higher energy and a larger rhodium–carbon distance than the intersection of the singlet curves, where the main photophysical channel leading to the rupture of the Rh-C_Me_ bond is opened. Even ignoring the importance of the gradient factor in the probability of this S/T transition, it can be assumed that this ISC pathway is most likely inactive. For Rh-C_Me_ distances greater than 2.30 Å, the lowest-energy singlet states, S_7_ and S_8_, intersect the curves of the states of T_1_–T_4_, as well as the T_34_ non-bonding state. In general, the calculated value of the SOC coefficient for the coupling of the S_7_ and S_8_ states with the T_1_-T_4_ states is not high and is in the range of 14 cm^−1^–34 cm^−1^. Moreover, the very flat curves of the singlet states are intersected by the curves of the triplet state at a relatively large angle; hence, the gradient coefficient at the curves’ intersection points will significantly reduce the probability of the S ⇝ T transition, and thus, the efficiency of the ISC process may be very limited. For the T_34_ state, the calculated SOCC is very large, equal to 533 cm^−1^. Such a high SOC coefficient value may indicate that the probability of occupying the triplet state is high and that a non-radiative transition to the triplet homolysis pathway would be possible during photodissociation along the singlet path. However, in the case of the S_7_ and S_8_ and T_34_ states, PEC intersections also occur at a large angle, which increases the gradient factor and thus limits the transition to the triplet state.

The interaction of the S_Soret_ singlet states (S_9_, S_10_) with the triplet states is very similar to the S_Q_ interactions with the T_9_, T_10_, and T_34_ states described above. The direct PEC intersection of the π → π* singlet states with the T_34_ σ → σ* state is particularly interesting. This intersection occurs at a slight elongation of the Rh-C_Me_ bond from the singlet states’ energy minimum and is associated with a very small energy barrier, approximately 2 kcal/mol. The SOCC calculated around the intersection point is ~108 cm^−1^. Due to the SOC coefficient, the probability of triplet state occupancy could be significant, but analogously to the S_Q_ states, the crossing of the S_9_ and S_10_ states with T_34_ occurs at a greater Rh-C_Me_ distance than the intersection of the PECs of the S_9_, S_10_/S_13_, and S_14_ singlet states. Therefore, the S_9_,S_10_/T_34_ ISC channel requires a larger stretching of the rhodium–carbon bond than the singlet IC channel. Although the bond length difference is not very large (0.5–1.0 Å), taking into account that IC processes are generally faster than ISC, it can be assumed that the occupation of the T_34_ σ → σ* state in such a photophysical pathway is rather inefficient. Likewise, the almost vertical slope of the T_34_ triplet curve relative to the S_9_ and S_10_ singlet curves decreases the probability of S/T transition between these states. The T_16_ and T_21_ triplet states are π → σ* states, while the T_19_ state is of d → σ* type. These excited states have a common feature, i.e., the donation of electronic density to the anti-bonding orbital of the Rh-C_Me_ bond. The PECs of these states cross the singlet state curves near their minimum, which means that in this case, the ISC process practically does not require overcoming an energy barrier. The calculated SOCCs for the interaction of these triplet states with S_Soret_ states for a distance R(Rh-C_Me_) = 2.00 Å range from 21 cm^−1^ for the S_9_/T_19_ coupling to 75 cm^−1^ for the S_9_/T_21_ and S_10_/T_21_ couplings. Although for some of the couplings the SOC coefficient is relatively large, the gradient factor may have, in this case, an unfavourable influence on the probability of S/T transitions due to the significant angle at which the curves of the triplet and singlet states intersect.

The computational results reveal that the direct ISC process between the S_Q_ and S_Soret_ states and the non-bonding triplet σ → σ* state is ineffective, which basically confirms the results of experimental studies indicating the lack of participation of triplet states in the photodissociation of the rhodium–carbon bond. However, it is difficult to rule out an indirect channel for the σ → σ* state population via other triplet states. Therefore, while such a possibility certainly exists, experimentally, geometry relaxation and internal conversion within the triplet states according to the Kasha rule lead exclusively to the occupation of the lowest T_1_ and T_2_ states. The T_1_ and T_2_ states are phosphorescent states and, simultaneously, they are not the initial states for the triplet homolysis of the rhodium–carbon bond. Meanwhile, from the point of view of the computational results, one could assume the existence of a dissociative triplet pathway: T_1_,T_2_ ⇝ T_9_,T_10_ ⇝ T_34_ ⇝ ^3^[RhPor ⋯ Me] ⇝ RhPor^↑^ + Me^↑^. The scope of the presented calculation results does not allow for the full solution to this problem; however, several reasons can be mentioned that may cause photophysical inactivity on the dissociative triplet path described above. Thus, it should be noted that in the case of photodissociation involving singlet states, the energies of the starting states S_Q_ and the energy of the products in the dissociation limit are comparable (Figure 6, S_1_ and S_2_ at R(Rh-C_Me_) = 2.00 Å vs. D_1_ and D_2_ levels at R(Rh-C_Me_) = ∞). The high energy of deexcitation of the S_1_ and S_2_ states to the S_0_ state, which according to calculations is 2.65 eV (61 kcal/mol), would correspond to a relatively short emission wave by fluorescence, 468 nm, but experimentally, fluorescence is not observed. It can therefore be assumed that despite the existence of an energy barrier in the photodissociation process, the rate constant for fluorescence is probably lower than for IC, which favours a dissociative path. In the case of triplet states, the opposite is true, i.e., the energy of the lowest triplet states T_1_ and T_2_ is lower than the energy of the products in the dissociation limit, and the total energy effect on the path T_1_ and T_2_ ⇝ RhPor^↑^ + Me^↑^ is associated with an increase in the system energy by ~6 kcal/mol. The computationally predicted maximum energy barrier on this path is 12 kcal/mol. Although this is not a high barrier for achieving the dissociated state, in the case of energetically unstable excited states, it may be the reason why the dissociative path is uncompetitive with respect to phosphorescence. It can, of course, be assumed that triplet photodissociation, despite everything, occurs to some very small extent, but the efficiency of this process is so low that it is difficult to observe it in an experiment.

The above discussion on singlet–triplet interactions involving the R(Rh-C_Me_) coordinate leads to the conclusion that most of the photophysical pathways related to ISC, which may occur via relaxation of the system in singlet states, are inactive or weakly active and do not contribute significantly to the triplet state population. It is worth noting, however, that the S/T interaction is possible for many closely lying triplet states around the photophysically active singlet states S_Q_ and S_Soret_. This can be evidenced by the calculated SOCC values for R(Rh-C_Me_) = 2.00 Å, which are varied but sometimes relatively large, even above 100 cm^−1^. For the vast majority of states, both singlet and triplet, the R(Rh-C_Me_) = 2.00 Å distance is, if not exactly, then essentially close to, the equilibrium geometry. In this context, for example, the SOCC for the interaction of the S_Soret_ states with the T_17_ triplet state of π/d → d character should be noted. The SOCC value for the coupling of the S_Soret_/T_17_ states is 200 cm^−1^ but quickly decreases to a level of several cm^−1^ with increasing rhodium–carbon distance. However, both in the case of the T_17_ state and many other triplet states located in the immediate vicinity of S_Q_ and S_Soret_, an alternative active coordinate for the S/T interaction could be the deformation of the equatorial coordination sphere of the complex. Such deformation is associated with the vibrational motion of the central ion along the axial axis of the complex and causes changes in the electronic structure of excited states (usually, it involves mixing of π and d orbitals). Consequently, this deformation may cause an increase in the value of the SOC integral and increase the probability of S ⇝ T conversion [55,56]. It should not be expected that even if such a mechanism of S/T interaction is taken into account, a dominant channel leading to triplet states will appear in the photophysics of MeRhPor, but probably for most pathways, ISC may be more effective in the case of coordination sphere deformation than in the case of rhodium–carbon bond elongation. Simultaneously, both the S ⇝ T ISC processes and the internal conversion between triplet states would proceed primarily along active coordinates other than the Rh-C_Me_ distance. The participation of different active coordinates in the ISC process may cause preference for specific photophysical paths, so in the case of triplet states, individual photophysical processes (ISC, IC, and geometric relaxation) that occur during the deformation of the coordination sphere may become more favourable for occupying the lowest triplet states and less advantageous for occupying potentially dissociative triplet states. The overall picture emerging from the computational results, regarding the contribution of triplet states to the photophysics of MeRhPor, seems to be that the population of the lowest phosphorescent triplet states is not the result of some dominant S_Q_ ⇝ T_1_,T_2_ and S_Soret_ ⇝ T_1_,T_2_ pathway but rather a composite of a larger number of individual and less effective S/T transitions. However, more elaborate consideration of this problem goes beyond the scope of this article and forms the basis for further theoretical studies and undoubtedly poses a challenge for experimental research.

## 4. Materials and Methods

The reported calculational results were obtained by applying the DFT [57,58,59] and TDDFT [60,61] level of theory with the use of the hybrid PBE0 functional [62,63], which mixes the Perdew–Burke–Ernzerhof (PBE) and HF exchange energy. The PBE0 hybrid functional is known as one of the effective functionals for describing the electronic structure of excited states of transition metal-containing systems and has been used for rhodoporphyrin complexes [33,64,65,66]. The def2-TZVP basis function [67] was applied for all atoms in the complex, albeit for rhodium, this basis utilises the effective core potential (ECP), replacing 28 core electrons [68]. In calculations, the RIJCOSX approximation [69,70] for the Coulomb and exchange parts of the Fock matrix was used, and the corresponding auxiliary basis sets were applied [71]. D3BJ dispersion corrections [72,73] were also employed in the calculations. To account for the interaction of the complex with the solvent environment, the continuous solvent model CPCM [74,75,76] with benzene as a solvent (ε = 2.28) was used in the calculations. The choice of solvent was due to the fact that the basic experimental data cited in the article [39] are from experiments for benzene solutions of methylrhodium(III)–octaethylporphyrin, MeRhOEP. For the calculation of the Rh-C_Me_ bond energy, the B3LYP [77,78], PBE [63], and BP86 [77,79] functionals were additionally used.

**Figure 12:**
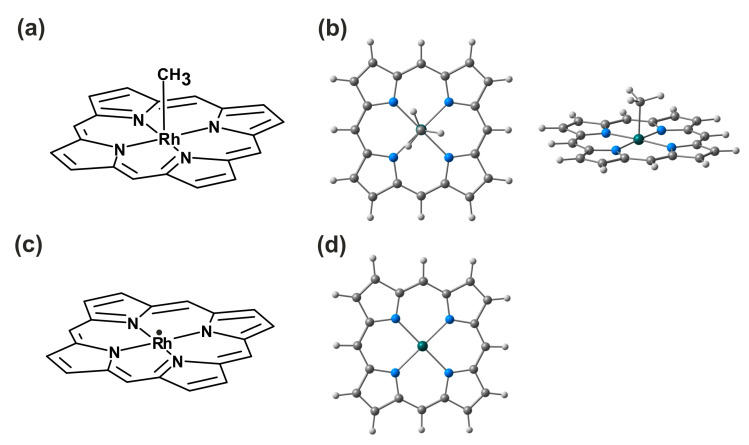
(**a**) Skeletal structure of MeRhPor (Me=CH_3_) and (**b**) structural model used in calculations; (**c**) skeletal structure of RhPor^•^ and (**d**) structural model used in calculations.

In the calculations, the model structure of methylrhodium(III)–porphyrin (MeRhPor) was used, in which the valence of the carbon atoms on the outer side of the macrocyclic ring is saturated with hydrogen atoms. The molecular structure of MeRhPor is shown in Figure 12. The Rh^+3^ ion is coordinated by a porphyrin ligand with a total charge of −2 through four nitrogen atoms present in four pyrrole subunits. The methyl group, formally with a charge of −1, coordinates the central ion through a carbon atom in the axial position. Thus, the MeRhPor structural model under consideration is a five-coordinate complex with a total charge of zero. The electronic ground state of MeRhPor is a low-spin singlet state, denoted as S_0_. Simultaneously, rhodium(II)–porphyrin (RhPor^•^), the product of homolytic photolysis of the Rh-C_Me_ bond, is a four-coordinate complex with a total charge of 0 and a D_0_ doublet ground state. For all molecular structures considered, it was formally assumed that these systems had no symmetry, and no symmetry options were used in the calculations.

By applying the restricted Kohn–Sham (RKS) and unrestricted Kohn–Sham (UKS) formalisms, respectively, the geometry of the MeRhPor complex was fully optimised for the two lowest electronic states, S_0_ and T_1_. Full geometry optimisation of the lowest excited singlet state S_1_ was performed using the TDDFT method. For the RhPor^•^ photolysis product, the geometry optimisation of the doublet ground state D_0_ was performed within the framework of the UKS formalism, while the geometry optimisation of the five lowest excited doublet states, D_1_–D_5_, was performed with a time-dependent variant of DFT.

For MeRhPor, to determine a PEC as a function of the Rh-C_Me_ distance, a scan of the S_0_ ground-state potential energy surface was performed in the range of 1.8 Å to 3.5 Å with a step of 0.05 Å. For individual PEC points, the Rh-C_Me_ distance was frozen, and the remaining parameters were fully optimised. Based on the obtained relaxed S_0_ state geometries, TDDFT calculations were performed for 50 singlet and triplet excited states. For the lowest triplet state T_1_, the potential energy curve was determined using the unrestricted KS method, optimising the geometry of the complex at particular frozen Rh-C_Me_ distances. In the calculations, both singlet and triplet pathways were examined to verify the experimental thesis. It is known that while the TDDFT method accurately captures the energies of singlet states, the triplet energies are somewhat less well reproduced [80]. A good description of triplet states is necessary in the calculations of many physical processes, including the TADF phenomenon [81,82,83]. Numerous benchmark calculations have been performed to verify this, showing that hybrid functionals perform better than non-hybrid functionals in this respect [81,82,83,84,85,86]. Using the Tamm–Dancoff approximation improves the quality of triplets [87]. However, the situation also strongly depends on the system being studied. Usually, the lowest triplet optimised by the UKS method provides the most reliable value for the energy of this state. Additionally, using the UKS formalism with a broken-symmetry wave function (BS WF), the PEC for the ground electronic state was determined in the full Rh-C_Me_ distance range from 1.80 Å to 3.50 Å.

To estimate the interactions between singlet and triplet electronic states obtained from TD-DFT and determine the values of SOC integrals, the formalism of the Quasi-Degenerate Perturbation Theory (QDPT) [88,89] was used. Since, essentially, the reliable estimation of the degree of spin–orbit coupling based on scalar relativistic methods requires the use of a full basis function, in this part of the calculations, the ZORA-def2-TZVP [67,90] and SARC-ZORA-TZVP [91] bases were applied for N, C, H, and Rh atoms. The ZORA-def2-TZVP basis is a relativistically recontracted version of the all-electron def2-TZVP Ahlrichs basis set, whereas the SARC-ZORA-TZVP basis set used for palladium is the segmented all-electron relativistically contracted (SARC) basis set. Calculations were performed using the geometry of the complex for the S_0_ electronic ground state, at PEC points corresponding to Rh-C_Me_ distances of 2.00 Å, 2.10 Å, 2.15 Å, and 2.30 Å. The geometry at these points was not re-optimised using the aforementioned basis sets. These points were chosen as characteristic of the interaction of singlet and triplet states in the photochemical process of elongation of the axial bond of rhodium–carbon.

Because MeRhPor is a structural model of the methylrhodium(III)–octaethylporphyrin complex (MeRhOEP), additional calculations were performed to check the influence of ethyl substituents on the structure and energetics of excited states. The results of the calculation are presented in Table S6 and Figure S8 and discussed in Supplementary Discussion S1 in the Supplementary Materials. Based on these results, it can be concluded that, according to the literature data [34,35], alkyl substituents at the β-pyrrolic position of the porphyrin ring have no significant effect on the energetics and electronic structure of the excited states of the rhodoporphyrin system.

All calculations were performed using the ORCA v. 5 package [92,93,94], with the SHARK package implemented for integral generation [95].

## 5. Conclusions

Under the influence of light with wavelengths in the Q and Soret band range, the MeRhPor complex molecule undergoes electronic excitation to singlet states, primarily S_1_ and S_2_ and S_9_ and S_10_ for the two absorption bands, respectively. All of these states are π → π* states, i.e., the electronic excitation involves the π orbitals of the porphyrin ligand. The excited states are deactivated in both singlet and triplet photophysical pathways. Singlet deactivation channels lead to homolytic scission of the axial rhodium–carbon bond. The excited states in the Soret band are deactivated via geometry relaxation and intersystem crossing (IC), which leads to a rapid scission of the Rh-C_Me_ bond. In this case, the states of π → σ*, π/d → π*, d → σ*, and π/d → σ* type participate indirectly in the photodecomposition of the MeRhPor complex. On the other hand, an energy barrier must be overcome from the excited states belonging to the Q band to reach the states leading directly to bond dissociation. Typically, these photodissociation states are characterised by the donation of electronic density to the σ* anti-bonding orbital of the bond that is being broken. As a result of photolytic decomposition, a methyl radical Me^•^ and a RhPor^•^ radical are formed. The methyl radical can react with molecules in the environment but could also, depending on the photoreaction conditions, partially recombine with the deactivated radical RhPor^•^. The lowest states of RhPor^•^ are thermally quenched via IC to the D_0_ state, in which the d_z2_ orbital of the rhodium ion is singly occupied. The RhPor^•^ radical in the electronic ground state D_0_ can recombine with the methyl radical and can react with molecules in the environment (e.g., O_2_) or form a two-centre dimer (RhPor)_2_. Ultimately, the reactions of the RhPor^•^ radical will be determined by the “external” conditions in which the photohomolysis reaction of the Rh-C_Me_ bond takes place. The population of the lowest triplet states, T_1_ and T_2_, is mediated by a series of higher triplets due to ISC processes with Q and Soret singlet states. In singlet–triplet interactions, there is probably no dominant ISC photophysical channel; instead, there are many more or less efficient intersystem crossing processes between the Q and Soret states and a series of closely lying triplet states. The lowest triplet states, T_1_ and T_2_, are quenched by phosphorescence. The inactivity of the triplet photodissociation channel is related to (a) the uncompetitiveness of the ISC leading to the direct occupancy of the dissociative triplet state relative to the photophysical channel IC of singlet states and (b) inefficient IC in the thermal transition T_1_ and T_2_ ⇝ ^3^[RhPor ⋯ Me] in relation to the radiative deactivation of the π → π* states T_1_ and T_2_.

For the MeRhPor molecule, quantum chemical calculations based on the DFT/TDDFT level of theory show a complex arrangement of closely spaced singlet as well as triplet states as a function of the Rh-C_Me_ distance, which indicates the possibility of different competing photophysical pathways. This property is not unique but is somehow accumulated in the methylrhodoporphyrin molecule. This leads to the belief that this system, as well as its analogues containing an axial rhodium–carbon bond, is an extremely “flexible” system from the perspective of photophysical and photochemical processes. Both the structure change within the axial bond and changes in the complex’s surroundings will affect the quantum yield of a specific process from the point of view of the deactivation of excited states.

## Figures and Tables

**Table 1:** Most important geometrical parameters of the coordination sphere for methylrhodium(III)–porphyrin complex (MeRhPor) and rhodium(II)–porphyrin (RhPor^•^) and calculated values of Rh-C_Me_ binding energy (ΔE_DE_), dissociation energy (ΔE_BDE_), and bond dissociation free energy (ΔG_BDFE_) in kcal/mol.

	**MeRhPor**				**RhPor^•^**			
	**S_0_**	**S_1_**	**T_1_**	**Exp. ^(^** **^a)^**	**S_0_**	**S_1_**	**S_3_**	**S_4_**
Bond length [Å]								
Rh-C_Me_	2.002	2.003	2.000	1.974				
Rh-N_21_	2.023	2.032	2.041	2.022	2.029	2.031	2.026	2.017
Rh-N_22_	2.023	2.030	2.041	2.033	2.028	2.005	2.026	2.017
Rh-N_23_	2.022	2.032	2.040	2.012	2.029	2.031	2.026	2.017
Rh-N_24_	2.023	2.030	2.042	2.044	2.028	2.005	2.026	2.017
Valence angle [°]								
N_21_-Rh-N_23_	177.3	177.3	178.2	178.8	180.0	180.0	180.0	180.0
N_22_-Rh-N_24_	177.2	177.2	178.2	178.5	180.0	180.0	180.0	180.0
Dihedral angle [°]								
N_21_-N_22_-N_23_-N_24_	0.0	0.1	0.0	0.2	0.0	0.0	0.0	0.0
N_21_-N_22_-N_23_-Rh	−1.9	−1.9	−1.3	−0.9	0.0	0.0	0.0	0.0
				[kcal/mol]				
					With dispersion correction			
	ΔE_DE_	ΔE_BDE_^(b)^	ΔG_BDFE_		ΔE_DE_	ΔE_BDE_^(b)^	ΔG_BDFE_	
PBE0	49.9	45.8	35.2		54.7	50.5	39.9	
B3LYP	46.7	42.6	32.1		54.5	50.3	39.8	
BP86	55.9	52.3	40.2		63.8	60.2	48.1	
PBE	59.0	54.5	44.1		63.8	59.2	48.6	
Exp.	58.0 ^(c)^	54.3 ^(d)^	41.0, 49.0 ^(e)^					

^(a)^ Ref. [40]. ^(b)^ Bond dissociation energy (BDE, ΔE_BDE_) is defined as bond energy (ΔE_DE_) with ZPE correction and thermal corrections. ^(c)^ Ref. [41]. ^(d)^ Ref. [42]. ^(e)^ Bond dissociation free energies (BDFEs) for Rh-C_Me_ bond: 41 kcal/mol and 49 kcal/mol are the BDFG values for the tetra(p-sulfonato-phenyl) porphyrin–rhodium complex in D_2_O and C_6_D_6_, respectively. Data from Ref. [43].

**Table 2:** Lowest vertical singlet electronic transitions for MeRhPor complex based on TDDFT/PBE0/def2-TZVP calculations with D3BJ dispersion correction and CPCM/benzene solvent model.

	**E (eV)**	**λ (nm)**	***f***	**%**	**Character**			**Experimental ^(a)^**
S_1_	2.68	463	0.0188	41	93 → 94	H → L	π_1_ → π_x_*	543 nm (2.28 eV)
				33	92 → 95	H-1 → L+1	π_2_ → π_y_*	
				14	93 → 95	H → L+1	π_1_ → π_y_*	
				11	92 → 94	H-1 → L	π_2_ → π_x_*	
S_2_	2.68	463	0.0188	41	93 → 95	H → L+1	π_1_ → π_y_*	
				33	92 → 94	H-1 → L	π_2_ → π_x_*	
				14	93 → 94	H → L	π_1_ → π_x_*	
				11	92 → 95	H-1 → L+1	π_2_ → π_y_*	
S_3_	2.83	438	0.0001	46	91 → 95	H-2 → L+1	π/d_xz_ → π_y_*	
				44	90 → 94	H-3 → L	π/d_yz_ → π_x_*	
S_4_	2.90	427	0.0000	46	90 → 95	H-3 → L+1	π/d_yz_ → π_y_*	
				46	91 → 94	H-2 → L	π/d_xz_ → π_x_*	
S_5_	2.96	419	0.0000	47	90 → 94	H-3 → L	π/d_yz_ → π_x_*	
				46	91 → 95	H-2 → L+1	π/d_xz_ → π_y_*	
S_6_	3.33	373	0.0053	42	90 → 95	H-3 → L+1	π/d_yz_ → π_y_*	
				42	91 → 94	H-2 → L	π/d_xz_ → π_x_*	
S_7_	3.40	365	0.0950	74	91 → 96	H-2 → L+2	π/d_xz_ → σ*	
S_8_	3.40	365	0.0967	74	90 → 96	H-3 → L+2	π/d_yz_ → σ*	
S_9_	3.75	330	1.5943	37	92 → 94	H-1 → L	π_2_ → π_x_*	395 nm (3.14 eV)
				32	93 → 95	H → L+1	π_1_ → π_y_*	
S_10_	3.75	330	1.6204	38	92 → 95	H-1 → L+1	π_2_ → π_y_*	
				32	93 → 94	H → L	π_1_ → π_x_*	
S_11_	3.79	327	0.0108	92	89 → 95	H-4 → L+1	d_x2-y2_ → π_y_*	
S_12_	3.79	327	0.0403	90	89 → 94	H-4 → L	d_x2-y2_ → π_x_*	

^(a)^ Experimental absorption wavelengths from Ref. [39].

## Data Availability

The raw data supporting the conclusions of this article will be made available by the authors on request.

## Supplementary Materials

The following supporting information can be downloaded at https://www.mdpi.com/article/10.3390/molecules30193855/s1: Supplementary Discussion: Supplementary Discussion 1. Simulated UV/VIS spectrum of MeRhOEP complex and character of excited singlet electronic states. Table S1: Selected, optimised geometrical parameters of the coordination sphere for methylrhodium(III)–porphyrin complex (MeRhPor) and rhodium(II)–porphyrin complex (RhPor•). Table S2: Natural transition orbitals (NTOs) for singlet electronic states of the MeRhPor complex. Table S3: The lowest vertical triplet electronic transitions for the MeRhPor complex based on the TD-DFT/PBE0/def2-TZVP calculations with D3BJ dispersion correction and the CPCM/benzene solvent model. Table S4: The lowest vertical electronic transitions for the RhPor^•^ complex based on the TD-DFT/ PBE0/def2-TZVP calculations with D3BJ dispersion correction and the CPCM/benzene solvent model. Table S5: Calculated values of spin–orbit coupling constant (SOCC) for selected states of the MeRhPor complex at certain Rh-C_Me_ distances. Table S6: The lowest vertical singlet electronic transitions for MeRhEOP (methylrhodium(III)–octaethylporphyrin) complex based on the TD-DFT/PBE0/def2-TZVP calculations with D3BJ dispersion correction and the CPCM/benzene solvent model. Figure S1: (a) Milliken spin population and (b) spin density isosurface (0.0035 a.u) of RhPor^•^ complex. Figure S2: Optimised structure of dimer (RhPor)_2_ and calculated bond energy (ΔE_DE_), bond dissociation energy (ΔE_BDE_), and bond dissociation free energy (ΔG_BDFE_) for Rh-Rh bond. Figure S3: Alpha and beta Kohn–Sham orbitals involved in the electronic excitations of the RhPor^•^ complex. Figure S4: Energy diagram of frontier Kohn–Sham orbitals for the RhPor^•^ complex. Figure S5: Energy diagram of Kohn–Sham frontier orbitals for two different Rh-C_Me_ bond lengths in the MeRhPor complex. Figure S6: Potential energy curves (PECs) as a function of the Rh-C_Me_ distance for the ground state (S_0_) and vertically excited singlet and triplet states of the MeRhPor complex. The potential energy curves for 21 singlet and 37 triplet states have been plotted on the basis of raw computational data obtained at the DFT and TDDFT levels of theory. Figure S7: Comparison of the potential energy curves of the ground state S_0_ and the lowest excited singlet state, obtained on the basis of calculations at the DFT, TDDFT, and CASSCF/NEVPT2 level of theory. Figure S8: Simulated UV/VIS spectra for the MeRhOEP complex and MeRhPor complex obtained on the basis of the TDDFT calculations [41,47,48,49,50,96,97].

## Abbreviations

The following abbreviations are used in this manuscript:

DFT	Density Functional Theory
TDDFT	Time-Dependent Density Functional Theory
MeRhPor	Methylrhodium(III)–porphyrin complex
RhPor^•^	Rhodium(II)–porphyrin radical
PEC	Potential energy curve
SOC	Spin–orbit coupling
SOCC	Spin–orbit coupling coefficient
IC	Internal conversion
ISC	Intersystem crossing
S_i_	i-th singlet electronic state
T_i_	i-th triplet electronic state
S_Q_	S_1_ and S_2_ electronic state
S_Soret_	S_9_ and S_10_ electronic state
NTOs	Natural transition orbitals
CASSCF/NEVPT2	Complete active space self-consistent field method in conjunction with the second order of perturbation theory in the N-Electron Valence State Perturbation Theory version.

## Appendix A

The transition probability between singlet and triplet electronic states can be treated as a Landau–Zener transition probability [51,52], defined by the formula

$$P_{ij}=\frac{-2\pi {\left|H_{ST}^{SO}\right|}^{2}}{\hbar F_{ST}\left|\frac{dR}{dt}\right|}$$

where

$$F_{ST}=\left|\frac{dE_{S}}{dR}-\frac{dE_{T}}{dR}\right|$$

$H_{ST}^{SO}$ is the spin–orbit coupling integral, $F_{ST}$ is the gradient factor, and *R* is the system active coordinate in the transition process between electronic states. $F_{ST}$ is defined as the difference in energy gradients at the intersection of the singlet and triplet electronic state surfaces. The S/T transition probability between the singlet and triplet states is proportional to the square of the modulus of the spin–orbital coupling integral (SOC coefficient, SOCC) and inversely proportional to the gradient factor and the change in the active coordinate over time.

## References

[1] Fleischer E.B. (1970). The Structure of Porphyrins and Metalloporphyrins. Acc. Chem. Res..

[2] Smith K.M. (1975). Porphyrins and Metalloporphyrins: A New Edition Based on the Original Volume by J. E. Falk.

[3] Kadish K.M., Smith K.M., Guilard R. (1999). The Porphyrin Handbook: Synthesis and Organic Chemistry.

[4] Momenteau M., Reed C.A. (1994). Synthetic Heme Dioxygen Complexes. Chem. Rev..

[5] Kim H.J., Khalimonchuk O., Smith P.M., Winge D.R. (2012). Structure, function, and assembly of heme centers in mitochondrial respiratory complexes. Biochim. Biophys. Acta.

[6] Mukherjee M. (2022). Heme Enzymes: Nature’s Versatile Catalysts. Am. J. Biomed. Sci. Res..

[7] Gao F., Jiaxuan Guo J., Yuanyue Shen Y. (2024). Advances from chlorophyll biosynthesis to photosynthetic adaptation, evolution and signaling. Plant Stress.

[8] Denisov I.G., Makris T.M., Sligar S.G., Schlichting I. (2005). Structure and Chemistry of Cytochrome P450. Chem. Rev..

[9] Wasielewski M.R. (1992). Photoinduced Electron Transfer in Supramolecular Systems for Artificial Photosynthesis. Chem. Rev..

[10] Lomova T., Tsaplev Y., Klyueva M., Ovchenkova E. (2021). Recent advances in the practical use of the redox properties of manganese porphyrins. J. Organomet. Chem..

[11] Cojocariu I., Carlotto S., Zamborlini G., Jugovac M., Schio L., Floreano L., Casarin M., Feyer V., Schneider C.M. (2021). Reversible redox reactions in metal-supported porphyrin: The role of spin and oxidation state. J. Mater. Chem. C.

[12] Sheldon R.A. (1994). Metalloporphyrins in Catalytic Oxidations.

[13] Kadish K.M., Smith K.M., Guilard R. (1999). The Porphyrin Handbook: Applications: Past, Present, and Future.

[14] Bonnett R. (1995). Photosensitizers of the porphyrin and phthalocyanine series for photodynamic therapy. Chem. Soc. Rev..

[15] Ogoshi H., Mizutani T. (1998). Multifunctional and Chiral Porphyrins: Model Receptors for Chiral Recognition. Acc. Chem. Res..

[16] Chandra R., Tiwari M., Kaur P., Sharma M., Jain R., Dass S. (2000). Metalloporphyrins-Application and Clinical Significance. Indian J. Clin. Biochem..

[17] Suslick K.S., Rakow N.A., Kosal M.E., Chou J.-H. (2000). The materials chemistry of porphyrins and metalloporphyrins. J. Porphyr. Phthalocyanines.

[18] Takagi S., Miharu Eguchi M., Donald A., Tryk D.A., Inoue H. (2006). Porphyrin photochemistry in inorganic/organic hybrid materials: Clays, layered semiconductors, nanotubes, and mesoporous materials. J. Photochem. Photobiol. C Photochem. Rev..

[19] Sekhar A.R., Chitose Y., Janoš J., Dangoor S.I., Ramundo A., Satchi-Fainaro R., Slavíček P., Klán P., Weinstain R. (2022). Porphyrin as a versatile visible-light-activatable organic/metal hybrid photoremovable protecting group. Nat. Commun..

[20] Ouyang J., Li D., Zhu L., Cai X., Liu L., Pan H., Ma A. (2024). Application and Challenge of Metalloporphyrin Sensitizers in Noninvasive Dynamic Tumor Therapy. Molecules.

[21] Imran M., Ramzan M., Qureshi A.K., Khan M.A., Tariq M. (2018). Emerging Applications of Porphyrins and Metalloporphyrins in Biomedicine and Diagnostic Magnetic Resonance Imaging. Biosensors.

[22] Boscencu R., Radulea N., Manda G., Machado I.F., Socoteanu R.P., Lupuliasa D., Burloiu A.M., Mihai D.P., Ferreira L.F.V. (2023). Porphyrin Macrocycles: General Properties and Theranostic Potential. Molecules.

[23] Brothers P.J., Collman J.P. (1986). The Organometallic Chemistry of Transition-Metal Porphyrin Complexes. Acc. Chem. Res..

[24] Wayland B.B., Sherry A.E., Coffin V.L. (2009). Homogeneous Transition Metal Catalyzed Reactions.

[25] Cui W., Wayland B.B. (2004). Hydrocarbon C-H bond activation by rhodium porphyrins. J. Porphyr. Phthalocyanines.

[26] de Bruin B., Hetterscheid D.G.H. (2007). Paramagnetic (Alkene)Rh and (Alkene)Ir Complexes: Metal or Ligand Radicals?. Eur. J. Inorg. Chem..

[27] Thompson S.J., Brennan M.R., Lee S.Y., Dong G. (2018). Synthesis and applications of rhodium porphyrin complexes. Chem. Soc. Rev..

[28] Campagna S., Puntoriero F., Nastasi F., Bergamini G., Balzani V., Balzani V., Campagna S. (2007). Photochemistry and Photophysics of Coordination Compounds: Ruthenium. Photochemistry and Photophysics of Coordination Compounds I. Topics in Current Chemistry.

[29] Bosch H.W., Wayland B.B. (1986). The role of rhodium porphyrins in the photoassisted formation of formaldehyde and methanol from hydrogen and carbon monoxide. Chem. Soc. Chem. Commun..

[30] Zhou J., Gai L., Mack J., Zhou Z., Qiu H., Chan K.S., Shen Z. (2016). Synthesis and photophysical properties of orthogonal rhodium(III)–carbon bonded porphyrin–aza-BODIPY conjugates. Mater. Chem. C.

[31] Yu M., Fu X. (2011). Visible Light Promoted Hydroxylation of a Si-C(sp^3^) Bond Catalyzed by Rhodium Porphyrins in Water. J. Am. Chem. Soc..

[32] Vasil’ev V.V., Borisov S.M., Golovina I.V. (2003). Luminescence of Water-Soluble Rh(III) Porphyrins. Opt. Spectrosc..

[33] Li H., Boao Han B., Wang R., Li W., Zhang W., Fu X., Fang H., Ma F., Wang Z., Zhang J. (2024). Photochemical conversion of CO to C1 and C2 products mediated by porphyrin rhodium(II) metallo-radical complexes. Nat. Commun..

[34] Kalyanasundaram K. (1984). Luminescence and triplet—Triplet absorption spectra of rhodium (III) porphyrins. Chem. Phys. Lett..

[35] Ogoshi H., Omura T., Yoshida Z. (1973). A New Rhodium(I)-Porphyrin Complex. II. Synthesis and Oxidative Alkylation. J. Am. Chem. Soc..

[36] Hanson L.K., Gouterman M., Hanson J.C. (1973). Porphyrins. XXIX. The Crystal and Molecular Structure and Luminescence of Bis(dimethylamine)etio(I)porphinatorhodium(III) Chloride Dihydrate. J. Am. Chem. Soc..

[37] Lever A.B.P., Ramaswamy B.S., Licoccia S. (1982). Sensitized photoreduction of methyl viologen by metalloporphyrins. J. Photochem..

[38] Hoshino M., Nagamori T., Seki H., Tase T., Chihara T., Lillis J.P., Wakatsuki Y. (1999). Laser Photolysis Studies on Photodissociation of Axial Ligands from Isocyanide Complexes of Cobalt(III) and Rhodium(III) Porphyrins in Toluene Solutions. A Comparison with the Photochemistry of Carbonylrhodium(III) Porphyrin. J. Phys. Chem. A.

[39] Hoshino M., Yasufuku K., Seki H., Yamazaki H. (1985). Wavelength-Dependent Photochemlcal Reaction of Methylrhodlum(III) Octaethylporphyrin. Studies on CH_3_-Rh Bond Cleavage. J. Phys. Chem..

[40] Whang D., Kim K. (1991). Structure of a new form of octaethylporphyrinato(methyl)rhodium(III). Acta Crystallogr. Sect. C Struct. Chem..

[41] Wayland B.B. (1998). Rh-Rh, Rh-H, Rh-C and Rh-O bond energies in (OEP)Rh complexes: Thermodynamic criteria for addition of M-H and M-M bonds to C-O and C-C multiple bonds. Polyhedron.

[42] Li G., Zhang F.F., Pi N., Chen H.L., Zhang S.Y., Chan K.S. (2001). Determination of Rh–C Bond Dissociation Energy in Methyl(porphyrinato)rhodium(III) Complexes: A New Application of Photoacoustic Calorimetry. Chem. Lett..

[43] Fu X., Wayland B.B. (2005). Thermodynamics of Rhodium Hydride Reactions with CO, Aldehydes, and Olefins in Water:  Organo-Rhodium Porphyrin Bond Dissociation Free Energies. J. Am. Chem. Soc..

[44] Gouterman M., Dolphin D. (1978). Optical Spectra and Electronic Structure of Porphyrins and Related Rings. The Porphyrins.

[45] Antipas A., Gouterman M. (1983). Porphyrins. 44. Electronic States of Co, Ni, Rh, and Pd Complexes. J. Am. Chem. Soc..

[46] Kuznetsov A.E. (2019). Stacks of Metalloporphyrins: Comparison of Experimental and Computational Results. J. Phys. Chem. B.

[47] Roos B.O., Lawley K.P. (1987). The Complete Active Space Self-Consistent Field Method and its Applications in Electronic Structure Calculations. Advances in Chemical Physics: Ab Initio Methods in Quantum Chemistry Part 2.

[48] Angeli C., Cimiraglia R., Evangelisti S., Leininger T., Malrieu J.-P. (2001). Introduction of n-electron valence states for multireference perturbation theory. J. Chem. Phys..

[49] Angeli C., Cimiraglia R., Malrieu J.-P. (2001). N-electron valence state perturbation theory: A fast implementation of the strongly contracted variant. Chem. Phys. Lett..

[50] Angeli C., Cimiraglia R., Malrieu J.-P. (2002). n-electron valence state perturbation theory: A spinless formulation and an efficient implementation of the strongly contracted and of the partially contracted variants. J. Chem. Phys..

[51] Landau L.D. (1932). Zur Theorie der Energieübertragung. II. Phys. Sov. Union.

[52] Zener C. (1932). Non-Adiabatic Crossing of Energy Levels. Proc. R. Soc. Lond. A..

[53] Thallmair S., Kowalewski M., Zauleck J.P.P., Roos M.K., de Vivie-Riedle R. (2014). Quantum Dynamics of a Photochemical Bond Cleavage Influenced by the Solvent Environment: A Dynamic Continuum Approach. J. Phys. Chem. Lett..

[54] Thallmair S., Zauleck J.P.P., de Vivie-Riedle R. (2015). Quantum Dynamics in an Explicit Solvent Environment: A Photochemical Bond Cleavage Treated with a Combined QD/MD Approach. J. Chem. Theory Comput..

[55] Szczepańska M., Lodowski P., Jaworska M. (2020). Electronic excited states and luminescence properties of palladium(II)corrin complex. J. Photochem. Photobiol. A Chem..

[56] Jaworska M., Lodowski P. Interaction of palladium porphyrin with dioxygen molecule. The perspective from theoretical calculation. Proceedings of the 6th EuChemS Inorganic Chemistry Conference.

[57] Hohenberg P., Kohn W. (1964). Inhomogeneous Electron Gas. Phys. Rev..

[58] Kohn W., Sham L.J. (1965). Self-Consistent Equations Including Exchange and Correlation Effects. Phys. Rev..

[59] Hohenberg P.C., Kohn W., Sham L.J. (1990). The Beginnings and Some Thoughts on the Future. Adv. Quantum Chem..

[60] Runge E., Gross E.K.U. (1984). Density-Functional Theory for Time-Dependent Systems. Phys. Rev. Lett..

[61] Casida M.E., Chong D.P. (1995). Time-Dependent Density Functional Response Theory for Molecules. Recent Advances in Density-Functional Methods.

[62] Adamo C., Barone V. (1999). Toward reliable density functional methods without adjustable parameters: The PBE0 model. J. Chem. Phys..

[63] Perdew J.P., Burke K., Ernzerhof M. (1996). Generalized Gradient Approximation Made Simple. Phys. Rev. Lett..

[64] Munro O.Q., Camp G.L., Carlton L. (2009). Structural, ^103^Rh NMR and DFT Studies of a Bis(phosphane)RhIII–Porphyrin Derivative. Eur. J. Inorg. Chem..

[65] Steinmetz M., Grimme S. (2013). Benchmark Study of the Performance of Density Functional Theory for Bond Activations with (Ni,Pd)-Based Transition-Metal Catalysts. ChemistryOpen.

[66] Maity B., Scott T.R., Stroscio G.D., Gagliardi L., Cavallo L. (2022). The Role of Excited States of LNi^II/III^(Aryl)(Halide) Complexes in Ni–Halide Bond Homolysis in the Arylation of C_sp3_–H Bonds. ACS Catal..

[67] Weigend F., Ahlrichs R. (2005). Balanced basis sets of split valence, triple zeta valence and quadruple zeta valence quality for H to Rn: Design and assessment of accuracy. Phys. Chem. Chem. Phys..

[68] Andrae D., Haeussermann U., Dolg M., Stoll H., Preuss H. (1990). Energy-adjustedab initio pseudopotentials for the second and third row transition elements. Theor. Chim. Acta.

[69] Neese F. (2003). An improvement of the resolution of the identity approximation for the formation of the Coulomb matrix. J. Comp. Chem..

[70] Neese F., Wennmohs F., Hansen A., Becker U. (2009). Efficient, approximate and parallel Hartree–Fock and hybrid DFT calculations. A ‘chain-of-spheres’ algorithm for the Hartree–Fock exchange. Chem. Phys..

[71] Weigend F. (2006). Accurate Coulomb-fitting basis sets for H to Rn. Phys. Chem. Chem. Phys..

[72] Grimme S., Ehrlich S., Goerigk L. (2011). Effect of the damping function in dispersion corrected density functional theory. J. Comput. Chem..

[73] Grimme S., Antony J., Ehrlich S., Krieg H. (2010). A consistent and accurate ab initio parametrization of density functional dispersion correction (DFT-D) for the 94 elements H-Pu. J. Chem. Phys..

[74] Marenich A.V., Cramer C.J., Truhlar D.G. (2009). Universal Solvation Model Based on Solute Electron Density and on a Continuum Model of the Solvent Defined by the Bulk Dielectric Constant and Atomic Surface Tensions. J. Phys. Chem. B.

[75] Garcia-Rates M., Neese F. (2019). Efficient implementation of the analytical second derivatives of hartree-fock and hybrid DFT energies within the framework of the conductor-like polarizable continuum model. J. Comput. Chem..

[76] Garcia-Rates M., Neese F. (2020). Effect of the Solute Cavity on the Solvation Energy and Its Derivatives Within the Framework of the Gaussian Charge Scheme. J. Comput. Chem..

[77] Becke A.D. (1988). Density-functional exchange-energy approximation with correct asymptotic-behavior. Phys. Rev. A.

[78] Lee C., Yang W., Parr R.G. (1988). Development of the Colle-Salvetti correlation-energy formula into a functional of the electron density. Phys. Rev. B.

[79] Perdew J.P. (1986). Density-functional approximation for the correlation-energy of the inhomogeneous electron-gas. Phys. Rev. B Condens. Matter Mater. Phys..

[80] Tozer D.J., Handy N.C. (2000). On the determination of excitation energies using density functional theory. Phys. Chem. Chem. Phys..

[81] Huang S., Zhang Q., Shiota Y., Nakagawa T., Kuwabara K., Yoshizawa K., Adachi C. (2013). Computational Prediction for Singlet- and Triplet-Transition Energies of Charge-Transfer Compounds. J. Chem. Theory Comput..

[82] Jacquemin D., Perpète E.A., Ciofini I., Adamo C. (2010). Assessment of Functionals for TD-DFT Calculations of Singlet−Triplet Transitions. J. Chem. Theory Comput..

[83] Wang J., Bai F.Q., Xia B.H., Zhang H.X., Cui T. (2014). Accurate simulation of geometry, singlet-singlet and triplet-singlet excitation of cyclometalated iridium(III) complex. J. Mol. Model..

[84] Bousquet D., Fukuda R., Jacquemin D., Ciofini I., Adamo C., Ehara M. (2014). Benchmark Study on the Triplet Excited-State Geometries and Phosphorescence Energies of Heterocyclic Compounds: Comparison Between TD-PBE0 and SAC-CI. J. Chem. Theory Comput..

[85] Atkins A.J., Talotta F., Freitag L., Boggio-Pasqua M., González L. (2017). Assessing Excited State Energy Gaps with Time-Dependent Density Functional Theory on Ru(II) Complexes. J. Chem. Theory Comput..

[86] Grotjahn R., Kaupp M. (2020). Validation of Local Hybrid Functionals for Excited States: Structures, Fluorescence, Phosphorescence, and Vibronic Spectra. J. Chem. Theory Comput..

[87] Rangel T., Hamed S.M., Bruneval F., J. Neaton J.B. (2017). An assessment of low-lying excitation energies and triplet instabilities of organic molecules with an ab initio Bethe-Salpeter equation approach and the Tamm-Dancoff approximation. J. Chem. Phys..

[88] Roemelt M., Maganas D., DeBeer S., Neese F. (2013). A combined DFT and restricted open-shell configuration interaction method including spin-orbit coupling: Application to transition metal L-edge X-ray absorption spectroscopy. J. Chem. Phys..

[89] de Souza B., Farias G., Neese F., Izsak R. (2019). Predicting Phosphorescence Rates of Light Organic Molecules Using Time-Dependent Density Functional Theory and the Path Integral Approach to Dynamics. J. Chem. Theory Comput..

[90] (2025). ORCA Manual, Version 6.0; Max-Planck-Institut für Kohlenforschung: Mülheim a. d. Ruhr, Germany. https://www.faccts.de/docs/orca/6.0/manual/.

[91] Rolfes J.D., Neese F., Pantazis D.A. (2010). All-electron scalar relativistic basis sets for the elements Rb–Xe. J. Comput. Chem..

[92] Neese F. (2012). The ORCA program system. WIRES Comput. Mol. Sci..

[93] Neese F., Wennmohs F., Becker U., Riplinger C. (2020). The ORCA quantum chemistry program package. J. Chem. Phys..

[94] Neese F. (2022). Software update: The ORCA program system, version 5.0. WIRES Comput. Mol. Sci..

[95] Neese F. (2023). The SHARK Integral Generation and Digestion System. J. Comput. Chem..

[96] Wayland B.B., Ba S., Sherry A.E. (1991). Activation of Methane and Toluene by Rhodium(II) Porphyrin Complexes. J. Am. Chem. Soc..

[97] Wayland B.B., Coffin V.L., Farnos M.D. (1988). Estimation of the Rh-Rh bond dissociation energy in the (octaethylporphyrina-to)rhodium(II) dimer by proton NMR line broadening. Inorg. Chem..

