# Supplementary material for: A Thorough Understanding of Methylrhodium(III)–Porphyrin Photophysics: A DFT/TDDFT Study
Source: Molecules. 2025 Sep 23;30(19):3855. doi: 10.3390/molecules30193855 (PMC12526158; doi:10.3390/molecules30193855)
Supplement: Supplementary file 1 [file molecules-30-03855-s001.zip › molecules-3842857-supplementary.pdf]

A Thorough Understanding of Methylrhodium(III)–porphyrin Photophysics: A DFT/TDDFT study

Piotr Lodowski, Maria Jaworska

Institute of Chemistry, University of Silesia in Katowice, Szkolna 9, PL 40-006 Katowice, Poland

Supplementary Materials

Supplementary Discussion.

Supplementary Discussion 1. Simulated UV/VIS spectrum of MeRhOEP complex and character of excited singlet electronic states.

Taking into account that the calculations were performed based on the MeRhPor structural model in which the ethyl substituents present at the porphyrin ring in the methylrhodium(III)–octaethylporphyrin complex (MeRhOEP) were replaced by hydrogen atoms, additional calculations were conducted to check the influence of ethyl substituents on the structure and energetics of excited states. Calculations were performed at the DFT/PBE0 level of theory using the same methodology as for the MeRhPor complex. For the ground-state optimised geometry of the MeRhOEP complex, calculations were performed for 50 vertically excited singlet states using the TDDFT method. The results of the calculation are shown in Table S5 and Figure S8. The shape of the simulated UV/VIS spectral line in the range of ~500 nm to ~330 nm is identical to the simulated spectral line for the MeRhPor complex, although the MeRhOEP spectrum is slightly shifted by about 10 nm towards longer wavelengths. A noticeable difference when comparing spectral lines is a slight change in the intensity of the Q and Soret bands. The identified Q band, including states S_1 and S_2, is slightly more intense compared to the analogous band for the MeRhPor complex. Simultaneously, the simulated Soret band, covering mainly excited states S_9 and S_{10}, has a slightly lower intensity compared to the simulated Soret band for MeRhPor. For the first thirteen excited states, corresponding to the electronic spectrum from the Q band to the Soret band, the character of the vertical excitations for the MeRhOEP complex fully coincides with the excitation characters of S_1 - S_{12} in the MeRhPor spectrum (Table S5 vs. Table 2 in the main text). Energetically, the average difference in the calculated excitation energies between the results for MeRhOEP and MeRhPro is 0.08 eV (~10 nm).

Supplementary Tables.

Table S1. Selected, optimised geometrical parameters of the coordination sphere for methylrhodium(III)-porphyrin complex (MeRhPor) and rhodium(II)-porphyrin complex (RhPor•).

	MeRhPor				RhPor•			
	S_0	S_1	T_1	Exp. [a)]	S_0	S_1	S_3	S_4
Bond length [Å]								
Rh-C_{Me}	2.002	2.003	2.000	1.974				
Rh-N_{21}	2.023	2.032	2.041	2.022	2.029	2.031	2.026	2.017
Rh-N_{22}	2.023	2.030	2.041	2.033	2.028	2.005	2.026	2.017
Rh-N_{23}	2.022	2.032	2.040	2.012	2.029	2.031	2.026	2.017
Rh-N_{24}	2.023	2.030	2.042	2.044	2.028	2.005	2.026	2.017
Valence angle [°]								
N_{21}-Rh-C_{Me}	92.0	91.8	91.5	90.7				
N_{22}-Rh-C_{Me}	89.8	90.0	89.3	89.2				
N_{23}-Rh-C_{Me}	90.7	90.8	90.3	90.5				
N_{24}-Rh-C_{Me}	93.0	92.9	92.5	92.3				
N_{21}-Rh- N_{22}	90.0	90.0	89.8	89.4	90.0	90.0	90.0	90.0
N_{22}-Rh- N_{23}	90.0	90.0	90.3	90.9	90.0	90.0	90.0	90.0
N_{23}-Rh- N_{24}	89.9	89.9	89.8	89.3	90.0	90.0	90.0	90.0
N_{24}-Rh- N_{21}	89.9	89.9	90.1	90.5	90.0	90.0	90.0	90.0
N_{21}-Rh- N_{23}	177.3	177.3	178.2	178.8	180.0	180.0	180.0	180.0
N_{22}-Rh- N_{24}	177.2	177.2	178.2	178.5	180.0	180.0	180.0	180.0
Dihedral angle [°]								
N_{21}-N_{22}-N_{23}-N_{24}	0.0	0.1	0.0	0.2	0.0	0.0	0.0	0.0
N_{21}-N_{22}-N_{23}-Rh	-1.9	-1.9	-1.3	-0.9	0.0	0.0	0.0	0.0

[a)] Whang, D.; Kim, K. Structure of a new form of octaethylporphyrinato(methyl)rhodium(III). *Acta Crystallographica Section C, Structural Chemistry* **1991**, *C47*, 2547-2550.

Table S2. Natural transition orbitals (NTOs) for singlet electronic states of the MeRhPor complex.

State	E(eV)	λ(nm)	Hole	Particle	*n*	Character
S_1	2.68	463	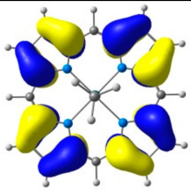	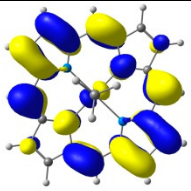	0.5527	$\pi \rightarrow \pi^*$
			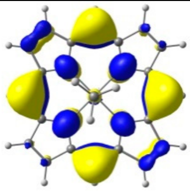	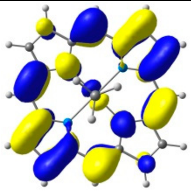	0.4409	$\pi \rightarrow \pi^*$
S_2	2.68	463	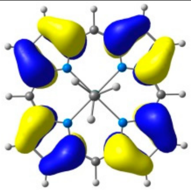	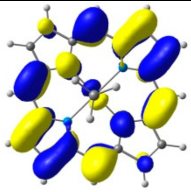	0.5525	$\pi \rightarrow \pi^*$
			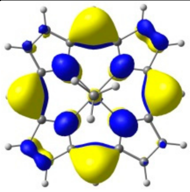	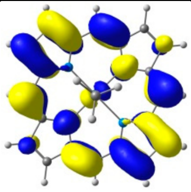	0.4411	$\pi \rightarrow \pi^*$
S_3	2.83	438	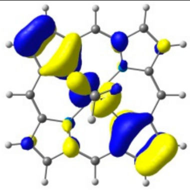	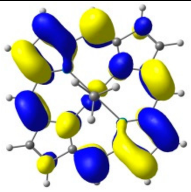	0.5087	$\pi/d \rightarrow \pi^*$
			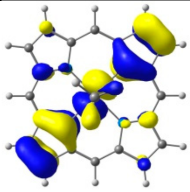	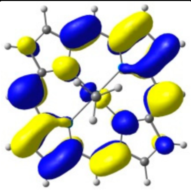	0.4881	$\pi/d \rightarrow \pi^*$
S_4	2.90	427	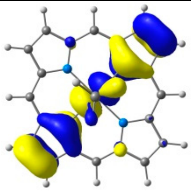	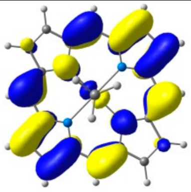	0.5056	$\pi/d \rightarrow \pi^*$
			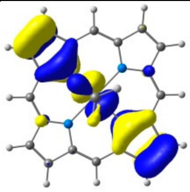	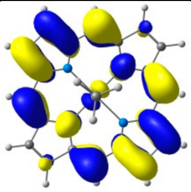	0.4876	$\pi/d \rightarrow \pi^*$

S_5	2.96	419	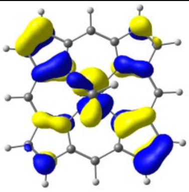	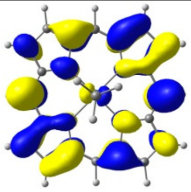	0.5022	π/d → π*
			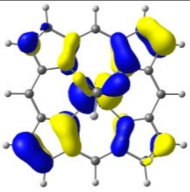	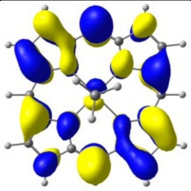	0.4900	π/d → π*
S_6	3.33	373	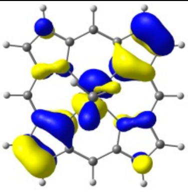	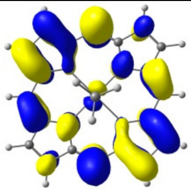	0.4670	π/d → π*
			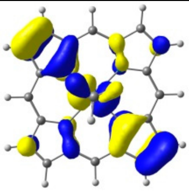	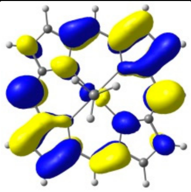	0.4609	π/d → π*
S_7	3.40	365	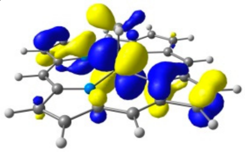	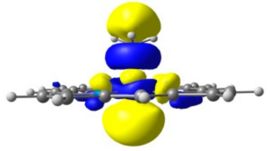	0.8694	π/d → σ*
S_8	3.40	365	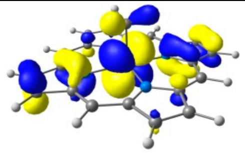	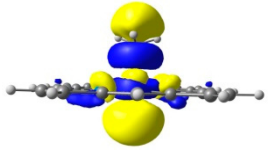	0.8679	π/d → σ*
S_9	3.75	330	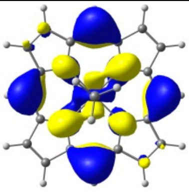	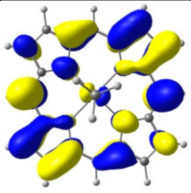	0.4441	π → π*
			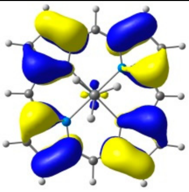	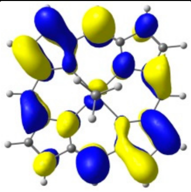	0.3714	π → π*
			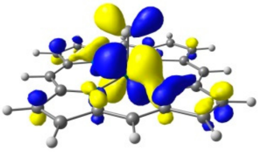	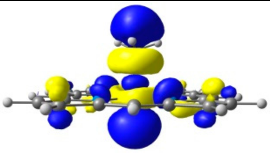	0.1330	d → σ*

S_{10}	3.75	330	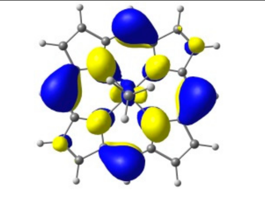	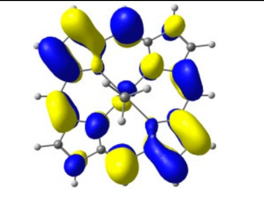	0.4333	$\pi \rightarrow \pi^*$
			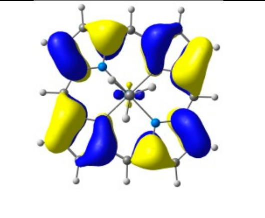	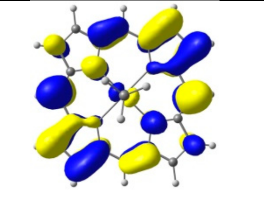	0.3763	$\pi \rightarrow \pi^*$
			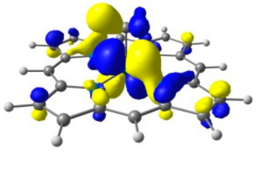	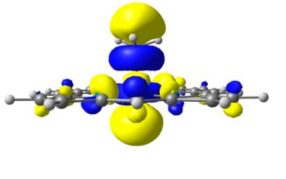	0.1368	$d \rightarrow \sigma^*$
S_{11}	3.79	327	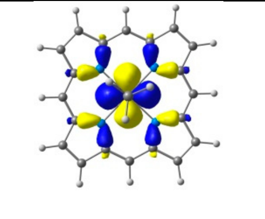	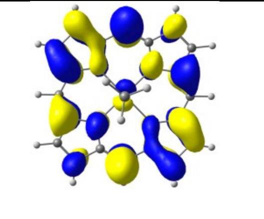	0.9666	$d \rightarrow \pi^*$
S_{12}	3.79	327	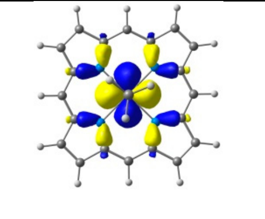	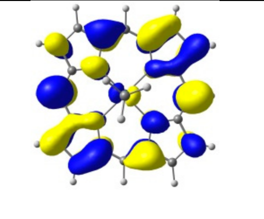	0.9963	$d \rightarrow \pi^*$

Table S3. The lowest vertical triplet electronic transitions for the MeRhPor complex based on the TD-DFT/PBE0/def2-TZVP calculations with D3BJ dispersion correction and the CPCM/benzene solvent model.

	E(eV)	λ(nm)	%	Character		
T_1	2.14	579	56	92 → 95	H-1 → L+1	$\pi_2 \rightarrow \pi_y^*$
			40	93 → 94	H → L	$\pi_1 \rightarrow \pi_x^*$
T_2	2.14	579	56	92 → 94	H-1 → L	$\pi_2 \rightarrow \pi_x^*$
			40	93 → 95	H → L+1	$\pi_1 \rightarrow \pi_y^*$
T_3	2.28	545	41	92 → 95	H-1 → L+1	$\pi_2 \rightarrow \pi_y^*$
			58	93 → 94	H → L	$\pi_1 \rightarrow \pi_x^*$
T_4	2.28	545	40	92 → 94	H-1 → L	$\pi_2 \rightarrow \pi_x^*$
			58	93 → 95	H → L+1	$\pi_1 \rightarrow \pi_y^*$
T_5	2.58	481	46	91 → 94	H-2 → L	$\pi/d_{xz} \rightarrow \pi_x^*$
			44	90 → 95	H-3 → L+1	$\pi/d_{yz} \rightarrow \pi_y^*$
T_6	2.64	469	45	91 → 95	H-2 → L+1	$\pi/d_{xz} \rightarrow \pi_y^*$
			43	90 → 94	H-3 → L	$\pi/d_{yz} \rightarrow \pi_x^*$
T_7	2.65	468	46	90 → 95	H-3 → L+1	$\pi/d_{yz} \rightarrow \pi_y^*$
			44	91 → 94	H-2 → L	$\pi/d_{xz} \rightarrow \pi_x^*$
T_8	2.70	460	47	90 → 94	H-3 → L	$\pi/d_{yz} \rightarrow \pi_x^*$
			45	91 → 95	H-2 → L+1	$\pi/d_{xz} \rightarrow \pi_y^*$
T_9	3.03	409	82	91 → 96	H-2 → L+2	$\pi/d_{xz} \rightarrow \sigma^*$
T_{10}	3.03	409	82	90 → 96	H-3 → L+2	$\pi/d_{yz} \rightarrow \sigma^*$
T_{11}	3.58	347	34	88 → 94	H-5 → L	$\pi \rightarrow \pi_x^*$
			25	88 → 95	H-5 → L+1	$\pi \rightarrow \pi_y^*$
			13	90 → 97	H-3 → L+3	$\pi/d_{yz} \rightarrow \pi^*$
			10	87 → 95	H-6 → L+1	$\pi \rightarrow \pi_y^*$
T_{12}	3.58	347	34	88 → 95	H-5 → L+1	$\pi \rightarrow \pi_y^*$
			25	88 → 94	H-5 → L	$\pi \rightarrow \pi_x^*$
			13	91 → 97	H-2 → L+3	$\pi/d_{xz} \rightarrow \pi^*$
			11	87 → 94	H-6 → L	$\pi \rightarrow \pi_x^*$
T_{13}	3.70	335	71	92 → 97	H-1 → L+3	$\pi_2 \rightarrow \pi^*$
T_{14}	3.73	333	76	89 → 95	H-4 → L+1	$d_{x2-y2} \rightarrow \pi_y^*$
			16	91 → 98	H-2 → L+4	$\pi/d_{xz} \rightarrow d_{xz}$-n
T_{15}	3.73	333	79	89 → 94	H-4 → L	$d_{x2-y2} \rightarrow \pi_x^*$
			14	90 → 98	H-3 → L+4	$\pi/d_{yz} \rightarrow d_{xz}$-n
T_{16}	3.76	330	91	92 → 96	H-1 → L+2	$\pi_2 \rightarrow \sigma^*$
T_{17}	3.78	328	65	91 → 98	H-2 → L+4	$\pi/d_{xz} \rightarrow d_{xz}$-n
			19	89 → 95	H-4 → L+1	$d_{x2-y2} \rightarrow \pi_y^*$
T_{18}	3.79	328	67	90 → 98	H-3 → L+4	$\pi/d_{yz} \rightarrow d_{xz}$-n
			16	89 → 94	H-4 → L	$d_{x2-y2} \rightarrow \pi_x^*$
T_{19}	3.82	325	94	89 → 96	H-4 → L+2	$d_{x2-y2} \rightarrow \sigma^*$
T_{20}	3.83	324	84	93 → 97	H → L+3	$\pi_1 \rightarrow \pi^*$
T_{34}	2.83	257	84	82 → 96	H-11 → L+2	$\sigma \rightarrow \sigma^*$

Table S4. The lowest vertical electronic transitions for the RhPor$^{\bullet}$ complex based on the TD-DFT/PBE0/def2-TZVP calculations with D3BJ dispersion correction and the CPCM/benzene solvent model.

	E(eV)	λ(nm)	*f*	S^2	%	Character		
D_1	0.42	2963	0.0000	0.76	80	$86\beta \rightarrow 89\beta$	H-2 → L	$\pi/d_{yz} \rightarrow d_{z2}$
					14	$78\beta \rightarrow 89\beta$	H-10 → L	$d_{yz}/\pi \rightarrow d_{z2}$
D_2	0.42	2949	0.0000	0.76	80	$85\beta \rightarrow 89\beta$	H-3 → L	$\pi/d_{xz} \rightarrow d_{z2}$
					14	$79\beta \rightarrow 89\beta$	H-9 → L	$d_{xz}/\pi \rightarrow d_{z2}$
D_3	1.17	1060	0.0000	0.76	97	$84\beta \rightarrow 89\beta$	H-4 → L	$d_{x2-y2} \rightarrow d_{z2}$
D_4	1.76	705	0.0012	0.77	99	$87\beta \rightarrow 89\beta$	H-1 → L	$\pi_2 \rightarrow d_{z2}$
D_5	1.85	672	0.0000	0.78	100	$88\beta \rightarrow 89\beta$	H → L	$\pi_1 \rightarrow d_{z2}$
D_6	2.12	586	0.0000	2.75	33	$88\alpha \rightarrow 91\alpha$	H-1 → L+1	$\pi_2 \rightarrow \pi_y^*$
					16	$89\alpha \rightarrow 90\alpha$	H → L	$\pi_1 \rightarrow \pi_x^*$
					32	$87\beta \rightarrow 91\beta$	H-1 → L+2	$\pi_2 \rightarrow \pi_y^*$
					15	$88\beta \rightarrow 90\beta$	H → L+1	$\pi_1 \rightarrow \pi_x^*$
D_7	2.12	586	0.0000	2.75	33	$88\alpha \rightarrow 90\alpha$	H-1 → L	$\pi_2 \rightarrow \pi_x^*$
					16	$89\alpha \rightarrow 91\alpha$	H → L+1	$\pi_1 \rightarrow \pi_y^*$
					32	$87\beta \rightarrow 90\beta$	H-1 → L+1	$\pi_2 \rightarrow \pi_x^*$
					15	$88\beta \rightarrow 91\beta$	H → L+2	$\pi_1 \rightarrow \pi_y^*$
D_8	2.26	549	0.0000	2.75	15	$88\alpha \rightarrow 91\alpha$	H-1 → L+1	$\pi_2 \rightarrow \pi_y^*$
					34	$89\alpha \rightarrow 90\alpha$	H → L	$\pi_1 \rightarrow \pi_x^*$
					16	$87\beta \rightarrow 91\beta$	H-1 → L+2	$\pi_2 \rightarrow \pi_y^*$
					32	$88\beta \rightarrow 90\beta$	H → L+1	$\pi_1 \rightarrow \pi_x^*$
D_9	2.26	549	0.0000	2.75	15	$88\alpha \rightarrow 90\alpha$	H-1 → L	$\pi_2 \rightarrow \pi_x^*$
					34	$89\alpha \rightarrow 91\alpha$	H → L+1	$\pi_1 \rightarrow \pi_y^*$
					16	$87\beta \rightarrow 90\beta$	H-1 → L+1	$\pi_2 \rightarrow \pi_x^*$
					32	$88\beta \rightarrow 91\beta$	H → L+2	$\pi_1 \rightarrow \pi_y^*$
D_{10}	2.64	469	0.0000	2.62	21	$86\beta \rightarrow 90\beta$	H-2 → L+1	$\pi/d_{yz} \rightarrow \pi_x^*$
					20	$85\beta \rightarrow 91\beta$	H-3 → L+2	$\pi/d_{xz} \rightarrow \pi_y^*$
					15	$85\beta \rightarrow 90\beta$	H-3 → L+1	$\pi/d_{xz} \rightarrow \pi_x^*$
					15	$86\beta \rightarrow 91\beta$	H-2 → L+2	$\pi/d_{yz} \rightarrow \pi_y^*$
D_{11}	2.66	466	0.0155	0.75	19	$88\beta \rightarrow 91\beta$	H → L+2	$\pi_1 \rightarrow \pi_y^*$
					18	$89\alpha \rightarrow 91\alpha$	H → L+1	$\pi_1 \rightarrow \pi_y^*$
					16	$88\alpha \rightarrow 90\alpha$	H-1 → L	$\pi_2 \rightarrow \pi_x^*$
					14	$87\beta \rightarrow 90\beta$	H-1 → L+1	$\pi_2 \rightarrow \pi_x^*$
					10	$88\beta \rightarrow 90\beta$	H → L+1	$\pi_1 \rightarrow \pi_x^*$
D_{12}	2.66	466	0.0155	0.75	19	$88\beta \rightarrow 90\beta$	H → L+1	$\pi_1 \rightarrow \pi_x^*$
					18	$89\alpha \rightarrow 90\alpha$	H → L	$\pi_1 \rightarrow \pi_x^*$
					16	$88\alpha \rightarrow 91\alpha$	H-1 → L+1	$\pi_2 \rightarrow \pi_y^*$
					14	$87\beta \rightarrow 91\beta$	H-1 → L+2	$\pi_2 \rightarrow \pi_y^*$
					10	$88\beta \rightarrow 91\beta$	H → L+2	$\pi_1 \rightarrow \pi_y^*$
D_{13}	2.69	460	0.0000	2.50	22	$85\beta \rightarrow 90\beta$	H-3 → L+1	$\pi/d_{xz} \rightarrow \pi_x^*$
					21	$86\beta \rightarrow 91\beta$	H-2 → L+2	$\pi/d_{yz} \rightarrow \pi_y^*$
					19	$85\beta \rightarrow 91\beta$	H-3 → L+2	$\pi/d_{xz} \rightarrow \pi_y^*$
					19	$86\beta \rightarrow 90\beta$	H-2 → L+1	$\pi/d_{yz} \rightarrow \pi_x^*$
D_{14}	2.75	450	0.0000	2.72	17	$86\beta \rightarrow 90\beta$	H-2 → L+1	$\pi/d_{yz} \rightarrow \pi_x^*$
					15	$85\beta \rightarrow 91\beta$	H-3 → L+2	$\pi/d_{xz} \rightarrow \pi_y^*$
					15	$86\beta \rightarrow 91\beta$	H-2 → L+2	$\pi/d_{yz} \rightarrow \pi_y^*$
					13	$85\beta \rightarrow 90\beta$	H-3 → L+1	$\pi/d_{xz} \rightarrow \pi_x^*$
					12	$86\alpha \rightarrow 91\alpha$	H-3 → L+1	$\pi/d_{xz} \rightarrow \pi_y^*$
					12	$87\alpha \rightarrow 90\alpha$	H-2 → L	$\pi/d_{yz} \rightarrow \pi_x^*$
D_{15}	2.77	447	0.0000	2.54	23	$85\beta \rightarrow 90\beta$	H-3 → L+1	$\pi/d_{xz} \rightarrow \pi_x^*$
					22	$86\beta \rightarrow 91\beta$	H-2 → L+2	$\pi/d_{yz} \rightarrow \pi_y^*$
					17	$85\beta \rightarrow 91\beta$	H-3 → L+2	$\pi/d_{xz} \rightarrow \pi_y^*$

					16	$86\beta \rightarrow 90\beta$	H-2 → L+1	$\pi/d_{yz} \rightarrow \pi_x$*
D_{16}	2.99	414	0.0000	1.01	25	$87\alpha \rightarrow 91\alpha$	H-2 → L+1	$\pi/d_{yz} \rightarrow \pi_y$*
					25	$86\alpha \rightarrow 90\alpha$	H-3 → L	$\pi/d_{xz} \rightarrow \pi_x$*
					15	$86\alpha \rightarrow 91\alpha$	H-3 → L+1	$\pi/d_{xz} \rightarrow \pi_y$*
					15	$87\alpha \rightarrow 90\alpha$	H-2 → L	$\pi/d_{yz} \rightarrow \pi_x$*
D_{17}	3.05	406	0.0000	0.89	20	$86\alpha \rightarrow 91\alpha$	H-3 → L+1	$\pi/d_{xz} \rightarrow \pi_y$*
					20	$87\alpha \rightarrow 90\alpha$	H-2 → L	$\pi/d_{yz} \rightarrow \pi_x$*
					16	$86\alpha \rightarrow 90\alpha$	H-3 → L	$\pi/d_{xz} \rightarrow \pi_x$*
					15	$87\alpha \rightarrow 91\alpha$	H-2 → L+1	$\pi/d_{yz} \rightarrow \pi_y$*
D_{18}	3.13	396	0.0000	0.97	22	$86\alpha \rightarrow 90\alpha$	H-3 → L	$\pi/d_{xz} \rightarrow \pi_x$*
					17	$86\alpha \rightarrow 91\alpha$	H-3 → L+1	$\pi/d_{xz} \rightarrow \pi_y$*
					17	$87\alpha \rightarrow 90\alpha$	H-2 → L	$\pi/d_{yz} \rightarrow \pi_x$*
					22	$87\alpha \rightarrow 91\alpha$	H-2 → L+1	$\pi/d_{yz} \rightarrow \pi_y$*
D_{19}	3.33	372	0.0000	0.78	99	$83\beta \rightarrow 89\beta$	H-5 → L	$\pi \rightarrow d_{z2}$
D_{20}	3.52	353	0.0000	0.79	19	$86\alpha \rightarrow 91\alpha$	H-3 → L+1	$\pi/d_{xz} \rightarrow \pi_y$*
					18	$87\alpha \rightarrow 90\alpha$	H-2 → L	$\pi/d_{yz} \rightarrow \pi_x$*
					11	$87\alpha \rightarrow 91\alpha$	H-2 → L+1	$\pi/d_{yz} \rightarrow \pi_y$*
					11	$86\alpha \rightarrow 90\alpha$	H-3 → L	$\pi/d_{xz} \rightarrow \pi_x$*
					10	$85\beta \rightarrow 91\beta$	H-3 → L+2	$\pi/d_{xz} \rightarrow \pi_y$*
					10	$86\beta \rightarrow 90\beta$	H-2 → L+1	$\pi/d_{yz} \rightarrow \pi_x$*
D_{21}	3.57	347	0.0027	2.75	27	$83\beta \rightarrow 90\beta$	H-5 → L+1	$\pi \rightarrow \pi_x$*
					26	$85\alpha \rightarrow 90\alpha$	H-4 → L	$\pi \rightarrow \pi_x$*
D_{22}	3.57	347	0.0027	2.75	27	$83\beta \rightarrow 91\beta$	H-5 → L+2	$\pi \rightarrow \pi_y$*
					26	$85\alpha \rightarrow 91\alpha$	H-4 → L+1	$\pi \rightarrow \pi_y$*
D_{23}	3.68	337	0.0000	2.75	37	$87\beta \rightarrow 92\beta$	H-1 → L+3	$\pi_2 \rightarrow \pi$*
					36	$88\alpha \rightarrow 92\alpha$	H-1 → L+2	$\pi_2 \rightarrow d_{xy}$-n
D_{24}	3.69	336	0.0000	1.88	48	$84\beta \rightarrow 90\beta$	H-4 → L+1	$d_{x2-y2} \rightarrow \pi_x$*
					48	$84\beta \rightarrow 91\beta$	H-4 → L+2	$d_{x2-y2} \rightarrow \pi_y$*
D_{25}	3.69	336	0.0000	1.88	48	$84\beta \rightarrow 90\beta$	H-4 → L+1	$d_{x2-y2} \rightarrow \pi_x$*
					48	$84\beta \rightarrow 91\beta$	H-4 → L+2	$d_{x2-y2} \rightarrow \pi_y$*
D_{26}	3.71	334	1.7705	0.76	23	$88\alpha \rightarrow 90\alpha$	H-1 → L	$\pi_2 \rightarrow \pi_x$*
					21	$87\beta \rightarrow 90\beta$	H-1 → L+1	$\pi_2 \rightarrow \pi_x$*
					19	$89\alpha \rightarrow 91\alpha$	H → L+1	$\pi_1 \rightarrow \pi_y$*
					19	$88\beta \rightarrow 91\beta$	H → L+2	$\pi_1 \rightarrow \pi_y$*
D_{27}	3.71	334	1.7692	0.76	23	$88\alpha \rightarrow 91\alpha$	H-1 → L+1	$\pi_2 \rightarrow \pi_y$*
					21	$87\beta \rightarrow 91\beta$	H-1 → L+2	$\pi_2 \rightarrow \pi_y$*
					19	$89\alpha \rightarrow 90\alpha$	H → L	$\pi_1 \rightarrow \pi_x$*
					19	$88\beta \rightarrow 89\beta$	H → L	$\pi_1 \rightarrow d_{z2}$
D_{28}	3.73	332	0.0000	0.81	59	$79\beta \rightarrow 89\beta$	H-9 → L	$d_{xz}/\pi \rightarrow d_{z2}$
					20	$78\beta \rightarrow 89\beta$	H-10 → L	$d_{yz}/\pi \rightarrow d_{z2}$
					12	$85\beta \rightarrow 89\beta$	H-3 → L	$\pi/d_{xz} \rightarrow d_{z2}$
D_{29}	3.73	332	0.0000	0.81	59	$78\beta \rightarrow 89\beta$	H-10 → L	$d_{yz}/\pi \rightarrow d_{z2}$
					20	$79\beta \rightarrow 89\beta$	H-9 → L	$d_{xz}/\pi \rightarrow d_{z2}$
					12	$86\beta \rightarrow 89\beta$	H-2 → L	$\pi/d_{yz} \rightarrow d_{z2}$
D_{30}	3.74	332	0.0030	0.78	98	$82\beta \rightarrow 89\beta$	H-6 → L	$\pi \rightarrow d_{z2}$

Table S5. Calculated values of spin-orbit coupling constant (SOCC) for selected states of the MeRhPor complex at certain Rh-C_{Me} distances.

States		SOC [cm^{-1}]						SOCC [cm^{-1}]	Triplet state character
Singlet	Triplet	Z		X		Y			
		ℜe	ℑm	ℜe	ℑm	ℜe	ℑm		
		R(Rh-C_{Me}) = 2.00 Å							
S_1	T_5	0	-22	0	3	0	22	31	π/d → π*
	T_6	0	10	0	-2	0	-32	34	π/d → π*
	T_7	0	-33	0	-7	0	-9	35	π/d → π*
	T_8	0	22	0	9	0	20	31	π/d → π*
	T_9	0	1	0	1	0	-1	2	π/d → σ*
	T_{10}	0	-1	0	2	0	-2	3	π/d → σ*
S_2	T_5	0	22	0	9	0	21	31	π/d → π*
	T_6	0	32	0	6	0	9	34	π/d → π*
	T_7	0	11	0	-7	0	-31	34	π/d → π*
	T_8	0	23	0	-4	0	-22	32	π/d → π*
	T_9	0	0	0	-2	0	3	4	π/d → σ*
	T_{10}	0	1	0	1	0	-2	2	π/d → σ*
S_9	T_{13}	0	-2	0	-3	0	-19	19	π → π*
	T_{14}	0	-1	0	6	0	0	6	d → π*
	T_{15}	0	-2	0	4	0	-2	5	d → π*
	T_{16}	0	16	0	9	0	49	52	π → σ*
	T_{17}	0	18	0	19	0	198	200	π/d → d
	T_{18}	0	6	0	-33	0	-17	38	π/d → d
	T_{19}	0	-3	0	-17	0	-8	19	d → σ*
	T_{20}	0	8	0	1	0	-2	9	π → π*
	T_{21}	0	67	0	6	0	-31	75	π → σ*
S_{10}	T_{13}	0	-15	0	-2	0	1	16	π → π*
	T_{14}	0	-7	0	6	0	3	9	d → π*
	T_{15}	0	-2	0	-10	0	-2	11	d → π*
	T_{16}	0	-17	0	-1	0	14	21	π → σ*
	T_{17}	0	191	0	29	0	-18	194	π/d → d
	T_{18}	0	-23	0	-20	0	4	31	π/d → d
	T_{19}	0	-15	0	36	0	-2	39	d → σ*
	T_{20}	0	0	0	2	0	-5	5	π → π*
	T_{21}	0	30	0	14	0	67	75	π → σ*
		R(Rh-C_{Me}) = 2.10 Å							
S_1	T_5	0	-24	0	2	0	24	34	π/d → π*
	T_6	0	-11	0	3	0	37	39	π/d → π*
	T_7	0	-37	0	-8	0	-10	39	π/d → π*
	T_8	0	25	0	10	0	23	35	π/d → π*
	T_9	0	0	0	-2	0	2	3	π/d → σ*
	T_{10}	0	2	0	-3	0	3	5	π/d → σ*
S_2	T_5	0	-24	0	-9	0	-23	34	π/d → π*
	T_6	0	37	0	7	0	10	39	π/d → π*
	T_7	0	-12	0	7	0	36	38	π/d → π*
	T_8	0	-26	0	3	0	25	35	π/d → π*
	T_9	0	0	0	-3	0	4	5	π/d → π*
	T_{10}	0	1	0	2	0	-2	3	π/d → σ*

S_{10}	T_{11}	0	-13	0	-16	0	-111	113	$\pi \rightarrow \sigma^*$
	T_{12}	0	25	0	-3	0	-71	75	$d \rightarrow \sigma^*$
	T_{13}	0	-1	0	3	0	-1	4	$\pi \rightarrow \pi^*$
	T_{14}	0	3	0	-7	0	-1	8	$\pi \rightarrow \pi^*$
	T_{15}	0	66	0	4	0	-48	82	$\pi \rightarrow \sigma^*$
	T_{16}	0	0	0	1	0	5	6	$\pi \rightarrow \pi^*$
	T_{17}	0	-3	0	-1	0	-2	4	$d \rightarrow \pi^*$
	T_{18}	0	0	0	-3	0	2	4	$d \rightarrow \pi^*$
	T_{19}	0	-7	0	14	0	-5	16	$\pi/d \rightarrow d$
	T_{20}	0	1	0	13	0	0	13	$\pi/d \rightarrow d$
	T_{21}	0	-1	0	3	0	-1	4	$\pi \rightarrow \pi^*$
S_{11}	T_{11}	0	-47	0	-12	0	-31	58	$\pi \rightarrow \sigma^*$
	T_{12}	0	-112	0	-16	0	6	113	$d \rightarrow \sigma^*$
	T_{13}	0	0	0	8	0	-1	8	$\pi \rightarrow \pi^*$
	T_{14}	0	-1	0	4	0	1	4	$\pi \rightarrow \pi^*$
	T_{15}	0	47	0	16	0	65	82	$\pi \rightarrow \sigma^*$
	T_{16}	0	3	0	1	0	1	3	$\pi \rightarrow \pi^*$
	T_{17}	0	5	0	-4	0	0	6	$d \rightarrow \pi^*$
	T_{18}	0	2	0	4	0	-3	5	$d \rightarrow \pi^*$
	T_{19}	0	2	0	12	0	-2	12	$\pi/d \rightarrow d$
	T_{20}	0	3	0	-20	0	-2	20	$\pi/d \rightarrow d$
		R(Rh-C_{Me}) = 2.15 Å							
S_1	T_5	0	-24	0	3	0	25	35	$\pi/d \rightarrow \pi^*$
	T_6	0	3	0	3	0	-3	5	$\pi/d \rightarrow \sigma^*$
	T_7	0	0	0	-6	0	4	7	$\pi/d \rightarrow \sigma^*$
	T_8	0	13	0	-3	0	-39	41	$\pi/d \rightarrow \pi^*$
	T_9	0	-38	0	-8	0	-11	41	$\pi/d \rightarrow \pi^*$
	T_{10}	0	26	0	10	0	23	36	$\pi/d \rightarrow \pi^*$
S_2	T_5	0	-25	0	-9	0	-23	35	$\pi/d \rightarrow \pi^*$
	T_6	0	2	0	7	0	-1	7	$\pi/d \rightarrow \sigma^*$
	T_7	0	-2	0	3	0	-3	5	$\pi/d \rightarrow \sigma^*$
	T_8	0	-39	0	-8	0	-12	41	$\pi/d \rightarrow \pi^*$
	T_9	0	-14	0	7	0	37	40	$\pi/d \rightarrow \pi^*$
	T_{10}	0	-26	0	3	0	26	37	$\pi/d \rightarrow \pi^*$
S_{11}	T_{11}	0	-22	0	-15	0	-88	92	$\pi \rightarrow \sigma^*$
	T_{12}	0	9	0	10	0	76	77	$d \rightarrow \sigma^*$
	T_{13}	0	-79	0	-8	0	30	85	$\pi \rightarrow \sigma^*$
	T_{14}	0	1	0	-6	0	1	6	$\pi \rightarrow \pi^*$
	T_{15}	0	-1	0	6	0	-1	6	$\pi \rightarrow \pi^*$
	T_{16}	0	2	0	-1	0	-4	5	$d \rightarrow \pi^*$
	T_{17}	0	0	0	1	0	3	3	$d \rightarrow \pi^*$
	T_{18}	0	-2	0	0	0	-3	4	$d \rightarrow \pi^*, \pi \rightarrow \pi^*$
	T_{19}	0	9	0	-9	0	21	24	$\pi/d \rightarrow d$
	T_{20}	0	1	0	4	0	-7	8	$\pi/d \rightarrow d$
	T_{21}	0	-36	0	-21	0	-99	107	$\sigma \rightarrow \sigma^*$
	T_{22}	0	-10	0	-2	0	2	10	$\pi \rightarrow \pi^*$
S_{12}	T_{11}	0	11	0	3	0	6	13	$\pi \rightarrow \sigma^*$
	T_{12}	0	-104	0	-14	0	21	107	$d \rightarrow \sigma^*$
	T_{13}	0	28	0	15	0	78	84.2	$\pi \rightarrow \sigma^*$
	T_{14}	0	-1	0	7	0	-1	6.9	$\pi \rightarrow \pi^*$
	T_{15}	0	-1	0	6	0	-1	6.6	$\pi \rightarrow \pi^*$
	T_{16}	0	3	0	1	0	0	3.6	$d \rightarrow \pi^*$
	T_{17}	0	0	0	6	0	7	9.4	$d \rightarrow \pi^*$

	T_{18}	0	1	0	1	0	2	2.5	d → π*, π → π*
	T_{19}	0	14	0	7	0	-6	16.7	π/d → d
	T_{20}	0	-6	0	17	0	-3	18.5	π/d → d
	T_{21}	0	-101	0	-10	0	39	108.3	σ → σ*
	T_{22}	0	-1	0	0	0	-7	7.0	π → π*
		R(Rh-C_{Me}) = 2.30 Å							
S_1 [a)]	T_5	0	-7	0	30	0	-6	31	π → π*
	T_6	0	2	0	-16	0	1	16	π → π*
	T_7	0	-36	0	8	0	112	118	π/d → π*
	T_8	0	69	0	2	0	-73	101	π/d → π*
	T_9	0	-60	0	-17	0	-56	84	π/d → π*
	T_{10}	0	103	0	20	0	32	109	π/d → π*
S_2 [a)]	T_5	0	0	0	-15	0	4	16	π → π*
	T_6	0	4	0	-30	0	5	31	π → π*
	T_7	0	-111	0	-21	0	-34	118	π/d → π*
	T_8	0	-71	0	-19	0	-67	100	π/d → π*
	T_9	0	-60	0	0	0	62	86	π/d → π*
	T_{10}	0	-33	0	6	0	104	109	π/d → π*
S_3 [b)]	T_7	0	-29	0	1	0	34	45	π/d → π*
	T_8	0	-16	0	3	0	43	46	π/d → π*
	T_9	0	-47	0	-10	0	-16	51	π/d → π*
	T_{10}	0	34	0	11	0	28	46	π/d → π*
	T_{11}	0	64	0	13	0	23	69	π/σ → σ*
S_4 [b)]	T_7	0	33	0	10	0	28	44	π/d → π*
	T_8	0	-43	0	-9	0	-15	46	π/d → π*
	T_9	0	18	0	-7	0	-47	51	π/d → π*
	T_{10}	0	30	0	-2	0	-34	45	π/d → π*
	T_{11}	0	-24	0	5	0	65	69	π/σ → σ*
		R(Rh-C_{Me}) = 2.40 Å							
S_1 [a)]	T_5	0	370	0	105	0	369	533	σ → σ*
	T_6	0	-19	0	-21	0	-20	34	π → π*
	T_7	0	15	0	0	0	15	21	π → π*
S_2 [a)]	T_5	0	378	0	11	0	-378	534	σ → σ*
	T_6	0	-22	0	3	0	21	31	π → π*
	T_7	0	17	0	-14	0	-13	25	π → π*
		R(Rh-C_{Me}) = 2.45 Å							
S_1 [a)]	T_4	0	-2	0	13	0	-2	14	π → π*
	T_5	0	1	0	-6	0	1	6	π → π*
	T_6	0	2	0	-12	0	1	12	π → π*
	T_7	0	1	0	-4	0	0	4	π → π*
S_2 [a)]	T_4	0	-1	0	6	0	-1	6	π → π*
	T_5	0	-2	0	13	0	-2	14	π → π*
	T_6	0	1	0	-4	0	0	4	π → π*
	T_7	0	-2	0	12	0	-1	12	π → π*

[a)] Singlet states S_1 and S_2 have π/d → σ* character at R(Rh-C_{Me}) = 2.30 Å.
[b)] Singlet states S_3 and S_4 have π → π* character at R(Rh-C_{Me}) = 2.30 Å.

Table S6. The lowest vertical singlet electronic transitions for the MeRhEOP (methylrhodium(III)-octaethylporphyrin) complex based on the TD-DFT/PBE0/def2-TZVP calculations with D3BJ dispersion correction and the CPCM/benzene solvent model.

	E(eV)	λ(nm)	*f*	%	Character			Experimental [a)]
1	2.61	476	0.0703	34	156 → 159	H-1 → L+1	$\pi_2 \rightarrow \pi_y$*	543 nm (2.28 eV)
				62	157 → 158	H → L	$\pi_1 \rightarrow \pi_x$*	
2	2.61	475	0.0618	35	156 → 158	H-1 → L	$\pi_2 \rightarrow \pi_x$*	
				61	157 → 159	H → L+1	$\pi_1 \rightarrow \pi_y$*	
3	2.76	449	0.0001	15	154 → 158	H-3 → L	$\pi/d_{yz} \rightarrow \pi_x$*	
				17	154 → 159	H-3 → L+1	$\pi/d_{yz} \rightarrow \pi_y$*	
				49	155 → 158	H-2 → L	$\pi/d_{xz} \rightarrow \pi_x$*	
				16	155 → 159	H-2 → L+1	$\pi/d_{xz} \rightarrow \pi_y$*	
4	2.82	439	0.0003	62	154 → 159	H-3 → L+1	$\pi/d_{yz} \rightarrow \pi_y$*	
				33	155 → 158	H-2 → L	$\pi/d_{xz} \rightarrow \pi_x$*	
5	2.89	428	0.0001	46	154 → 158	H-3 → L	$\pi/d_{yz} \rightarrow \pi_x$*	
				49	155 → 159	H-2 → L+1	$\pi/d_{xz} \rightarrow \pi_y$*	
6	3.22	386	0.0049	31	154 → 158	H-3 → L	$\pi/d_{yz} \rightarrow \pi_x$*	
				17	154 → 159	H-3 → L+1	$\pi/d_{yz} \rightarrow \pi_y$*	
				15	155 → 158	H-2 → L	$\pi/d_{xz} \rightarrow \pi_x$*	
				31	155 → 159	H-2 → L+1	$\pi/d_{xz} \rightarrow \pi_y$*	
7	3.29	377	0.0738	76	155 → 160	H-2 → L+2	$\pi/d_{xz} \rightarrow \sigma$*	
8	3.30	375	0.0834	75	154 → 160	H-3 → L+2	$\pi/d_{yz} \rightarrow \sigma$*	
9	3.64	341	1.3860	10	153 → 158	H-4 → L	$\pi \rightarrow \pi_x$*	395 nm (3.14 eV)
				38	156 → 159	H-1 → L+1	$\pi_2 \rightarrow \pi_y$*	
				23	157 → 158	H → L	$\pi_1 \rightarrow \pi_x$*	
10	3.65	340	1.4344	10	153 → 158	H-4 → L	$\pi \rightarrow \pi_x$*	
				38	156 → 158	H-1 → L	$\pi_2 \rightarrow \pi_x$*	
				24	157 → 159	H → L+1	$\pi_1 \rightarrow \pi_y$*	
11	3.65	339	0.0068	99	157 → 160	H → L+2	$\pi_1 \rightarrow \sigma$*	
12	3.82	325	0.0047	86	151 → 158	H-6 → L	$d_{x2-y2} \rightarrow \pi_x$*	
13	3.83	324	0.0044	85	151 → 159	H-6 → L+1	$d_{x2-y2} \rightarrow \pi_y$*	
				11	152 → 159	H-5 → L+1	$\pi/d_{x2-y2} \rightarrow \pi_y$*	

[a)] Experimental values from Hoshino, M.; Yasufuku, K.; Seki, H.; Yamazaki, H. Wavelength-Dependent Photochemlcal Reaction of Methylrhodlum(III) Octaethylporphyrin. Studies on CH_3-Rh Bond Cleavage. *J. Phys. Chem.* **1985**, *89*, 3080-3085.

Supplementary Figures.

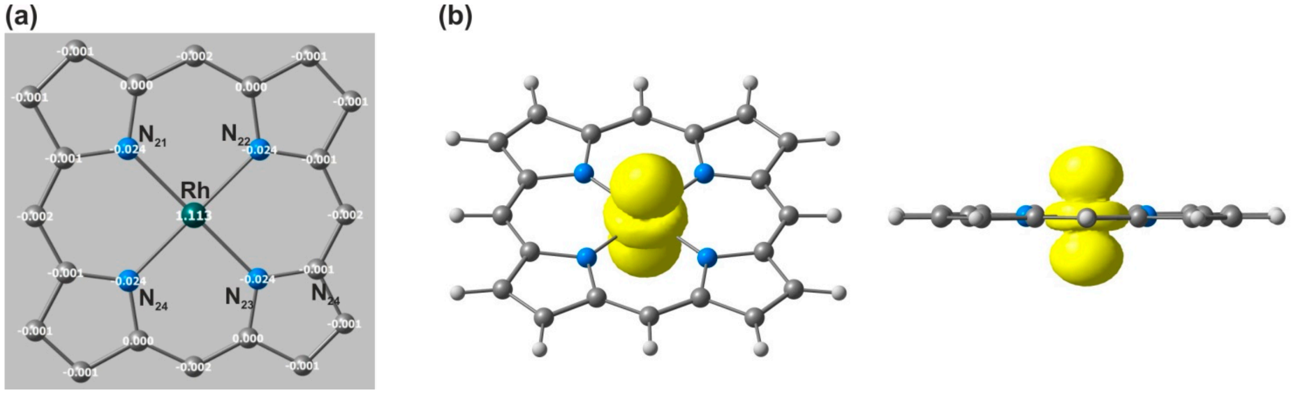

Figure S1. (a) Milliken spin population and (b) spin density isosurface (0.0035 a.u) of RhPor$^{\bullet}$ complex.

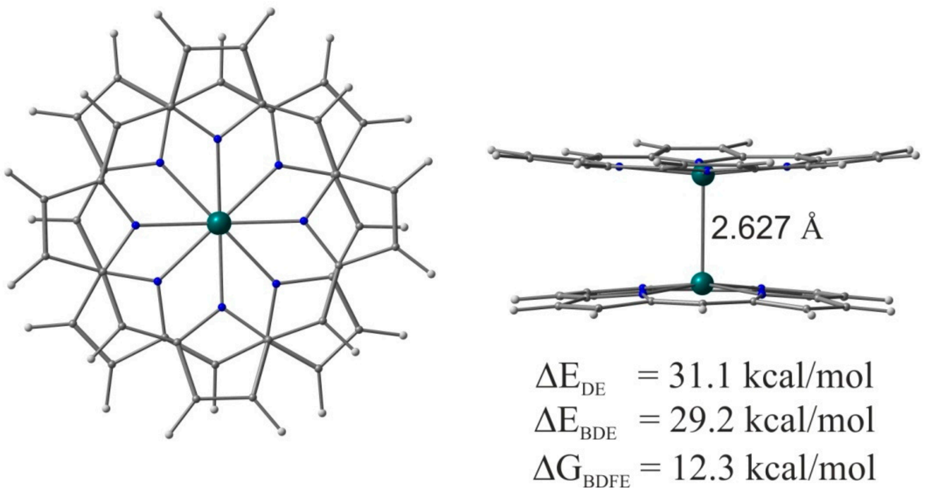

Figure S2. Optimised structure of dimer $(RhPor)_2$ and calculated bond energy (ΔE_{DE}), bond dissociation energy (ΔE_{BDE}), and bond dissociation free energy (ΔG_{BDFE}) for Rh-Rh bond. The experimental BDE for the Rh-Rh bond is about 10 – 20 kcal/mol. (Wayland, B. B.; Ba, S.; Sherry, A. E. Activation of Methane and Toluene by Rhodium(II) Porphyrin Complexes. *J. Am. Chem. Soc.* **1991**, *113*, 5305–5311. Wayland, B. B. Rh-Rh, Rh-H, Rh-C and Rh-O bond energies in (OEP)Rh complexes: Thermodynamic criteria for addition of M-H and M-M bonds to C-O and C-C multiple bonds. *Polyhedron* **1988**, 7, 1545–1555. Wayland, B. B.; Coffin, V. L.; Farnos, M. D. Estimation of the Rh-Rh bond dissociation energy in the (octaethylporphyrinato)rhodium(II) dimer by proton NMR line broadening. *Inorg. Chem.* **1988**, *27*, 2745–2747.)

Alpha Kohn-Sham orbitals

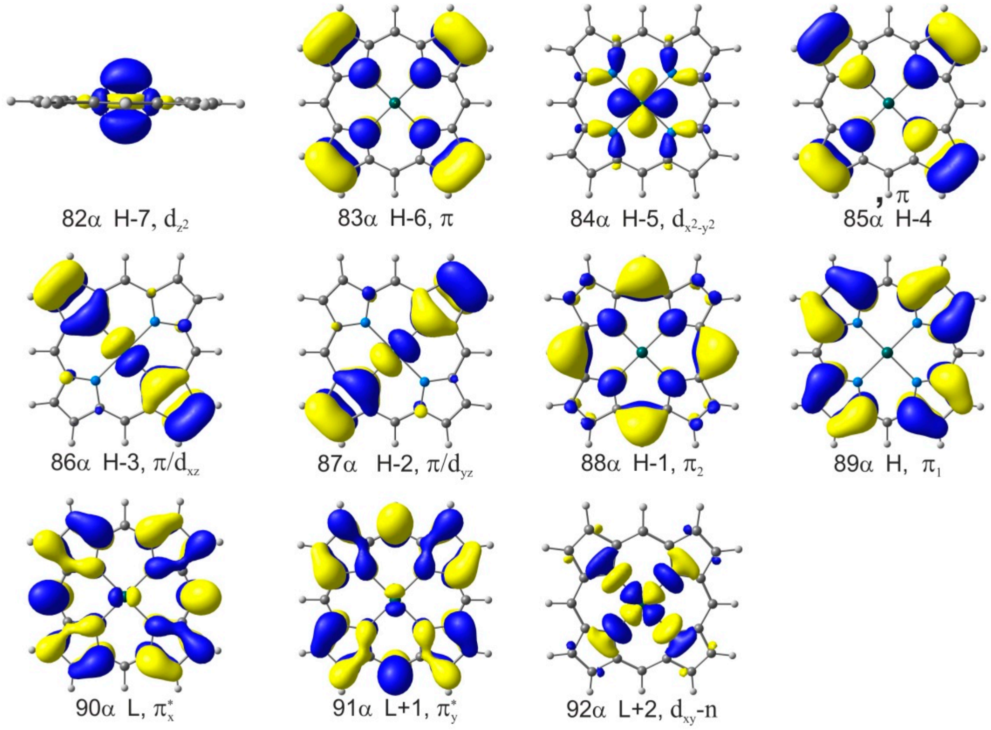

Beta Kohn-Sham orbitals

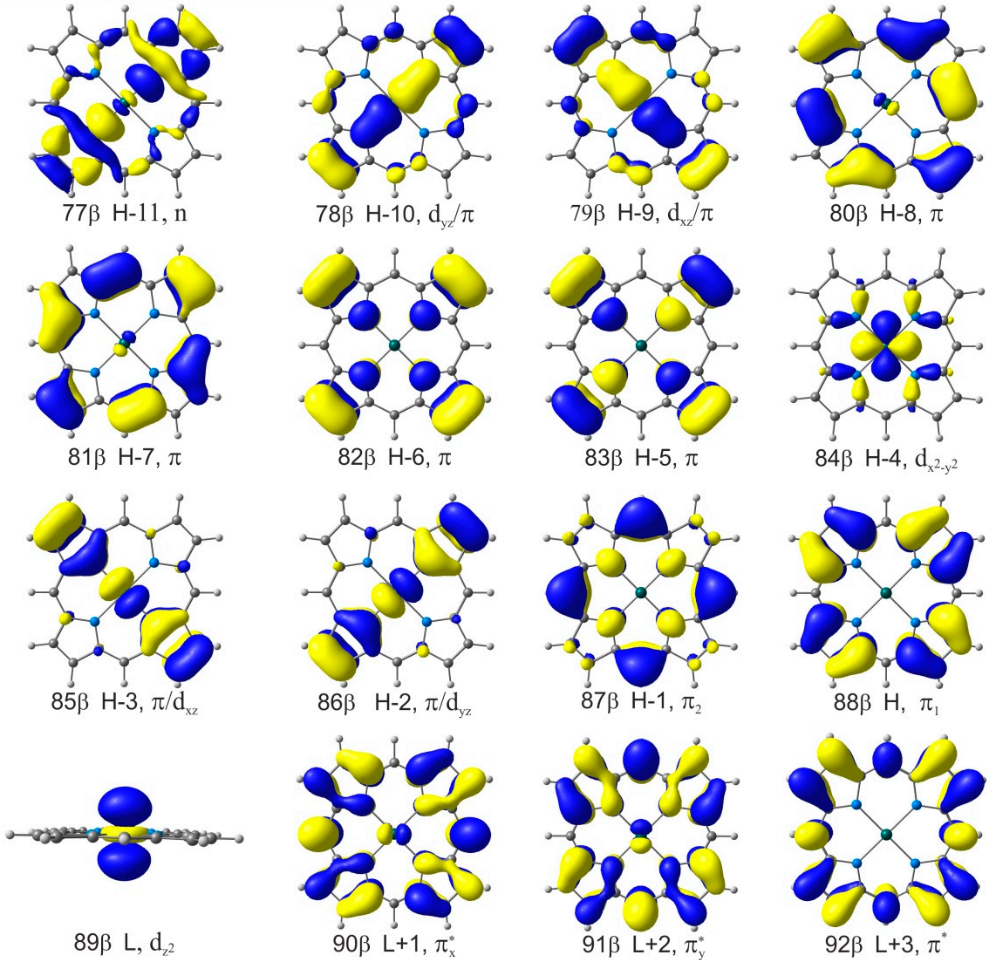

Figure S3. Alpha and beta Kohn–Sham orbitals involved in the electronic excitations of the RhPor• complex.

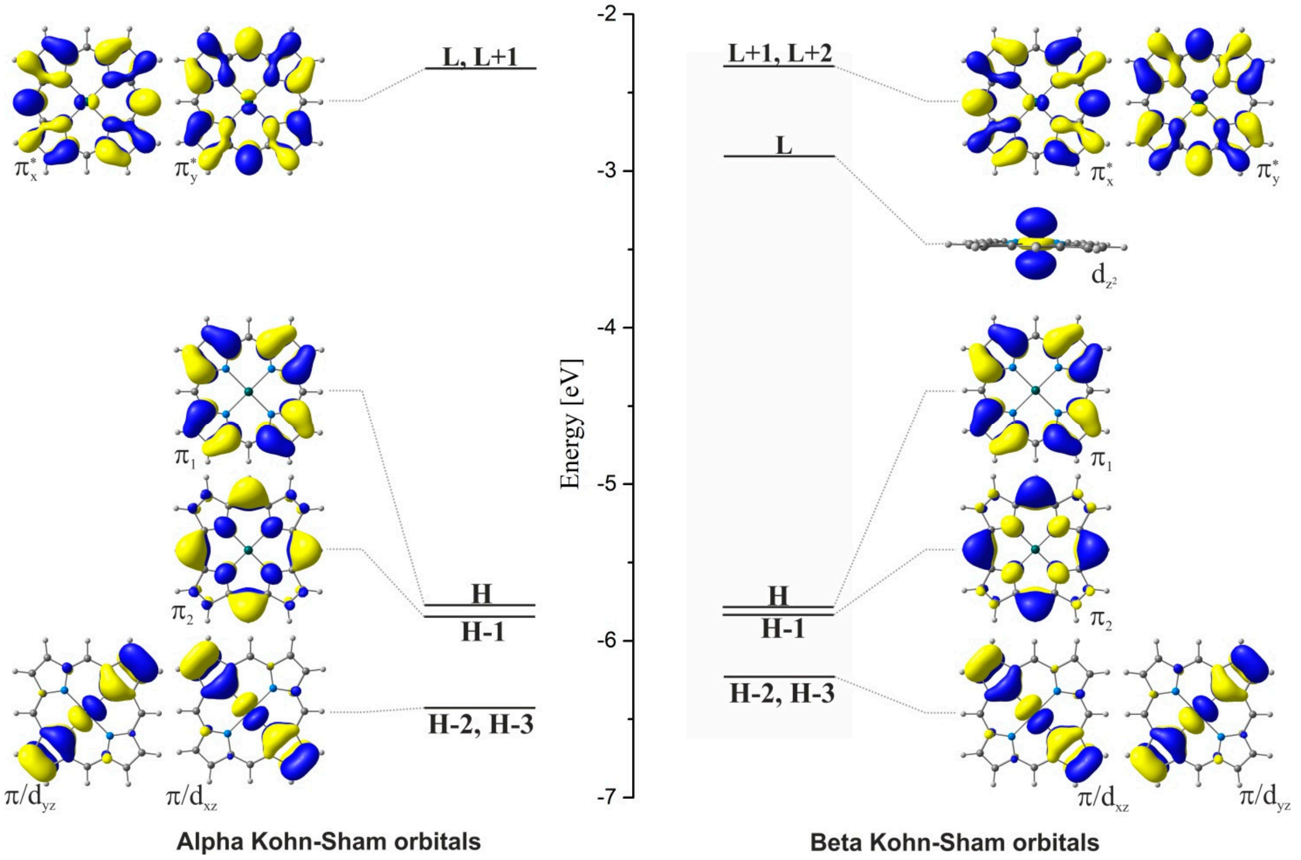

Figure S4. Energy diagram of frontier Kohn–Sham orbitals for the RhPor• complex.

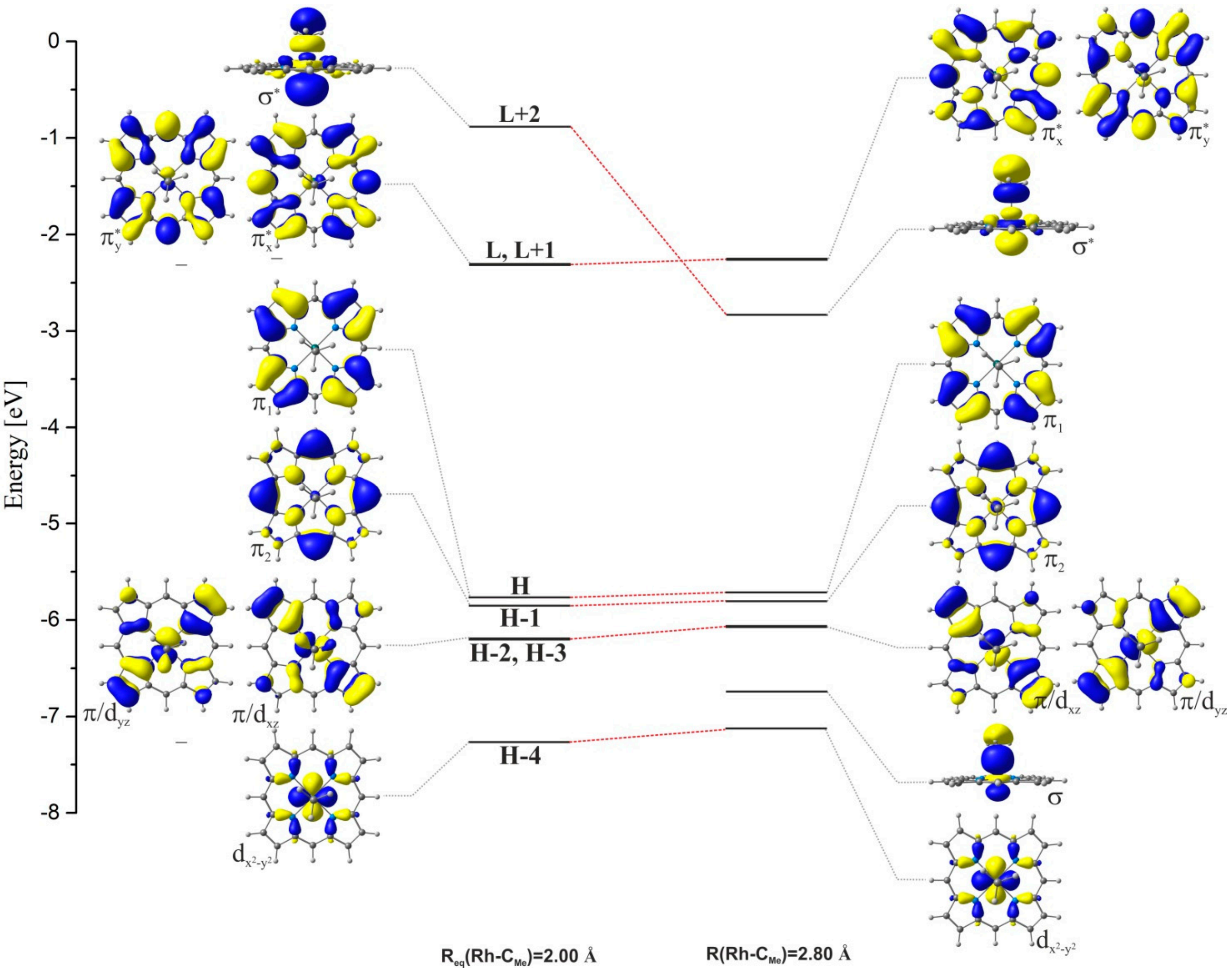

Figure S5. Energy diagram of Kohn–Sham frontier orbitals for two different Rh-C_{Me} bond lengths in the MeRhPor complex.

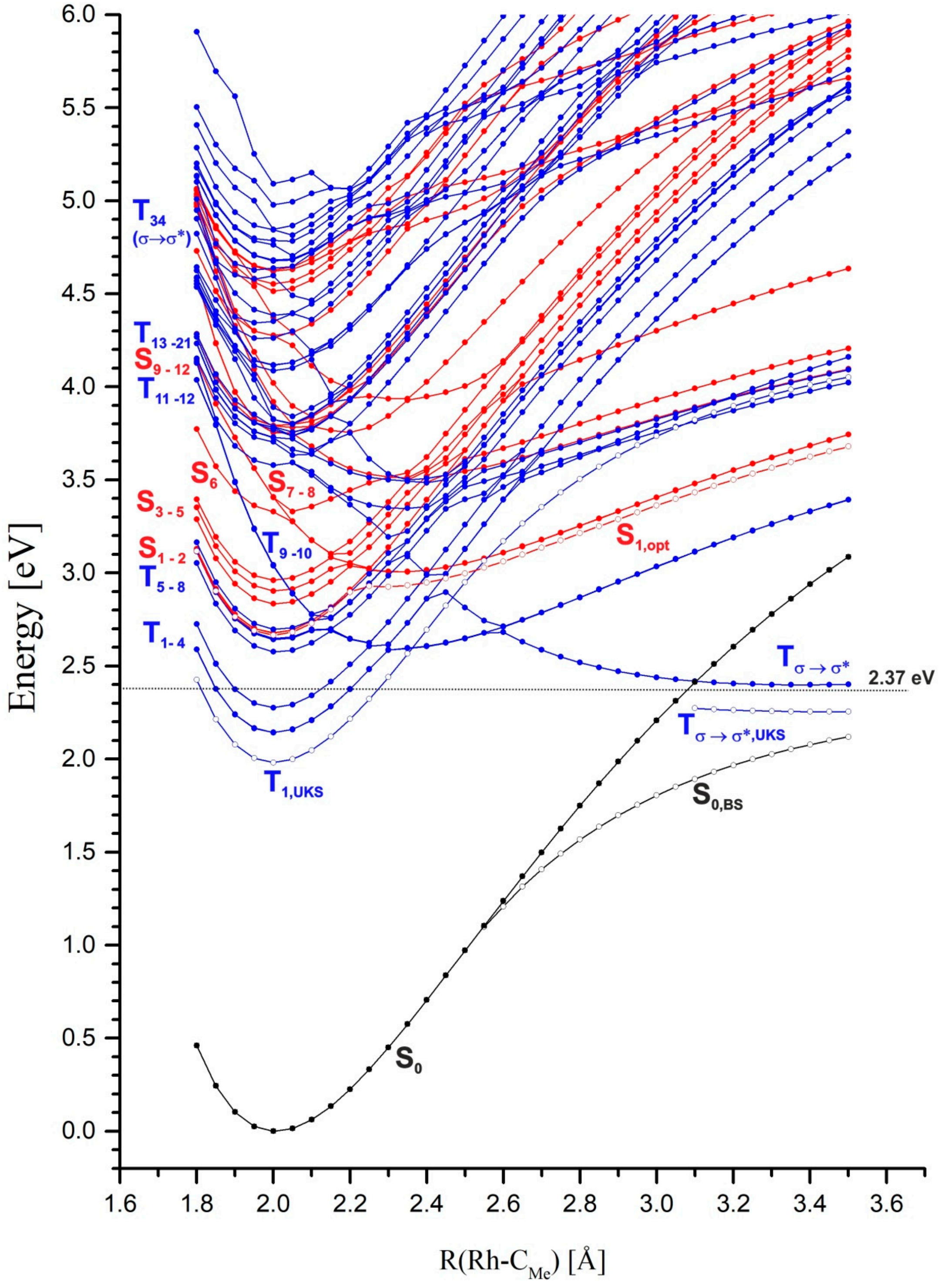

Figure S6. Potential energy curves (PECs) as a function of the Rh-C_{Me} distance for the ground state (S_0) and vertically excited singlet and triplet states of the MeRhPor complex. The potential energy curves for 21 singlet and 37 triplet states have been plotted on the basis of raw computational data obtained at the DFT and TDDFT levels of theory. Black line—PEC of S_0 state obtained from restricted Kohn–Sham method (RKS); red lines - PECs of singlet excited states from TDDFT level of theory; blue lines - PECs of triplet excited states from TDDFT level of theory; black line with empty circles - PEC of ground state, $S_{0,BS}$, obtained from broken-symmetry (BS) wave function; blue line with empty circles - PEC of lowest triplet state $T_{1,UKS}$, obtained from unrestricted Kohn–Sham method (UKS); red line with empty circles - PEC of first excited singlet state $S_{1,opt}$ obtained for optimised geometry at TDDFT level of theory.

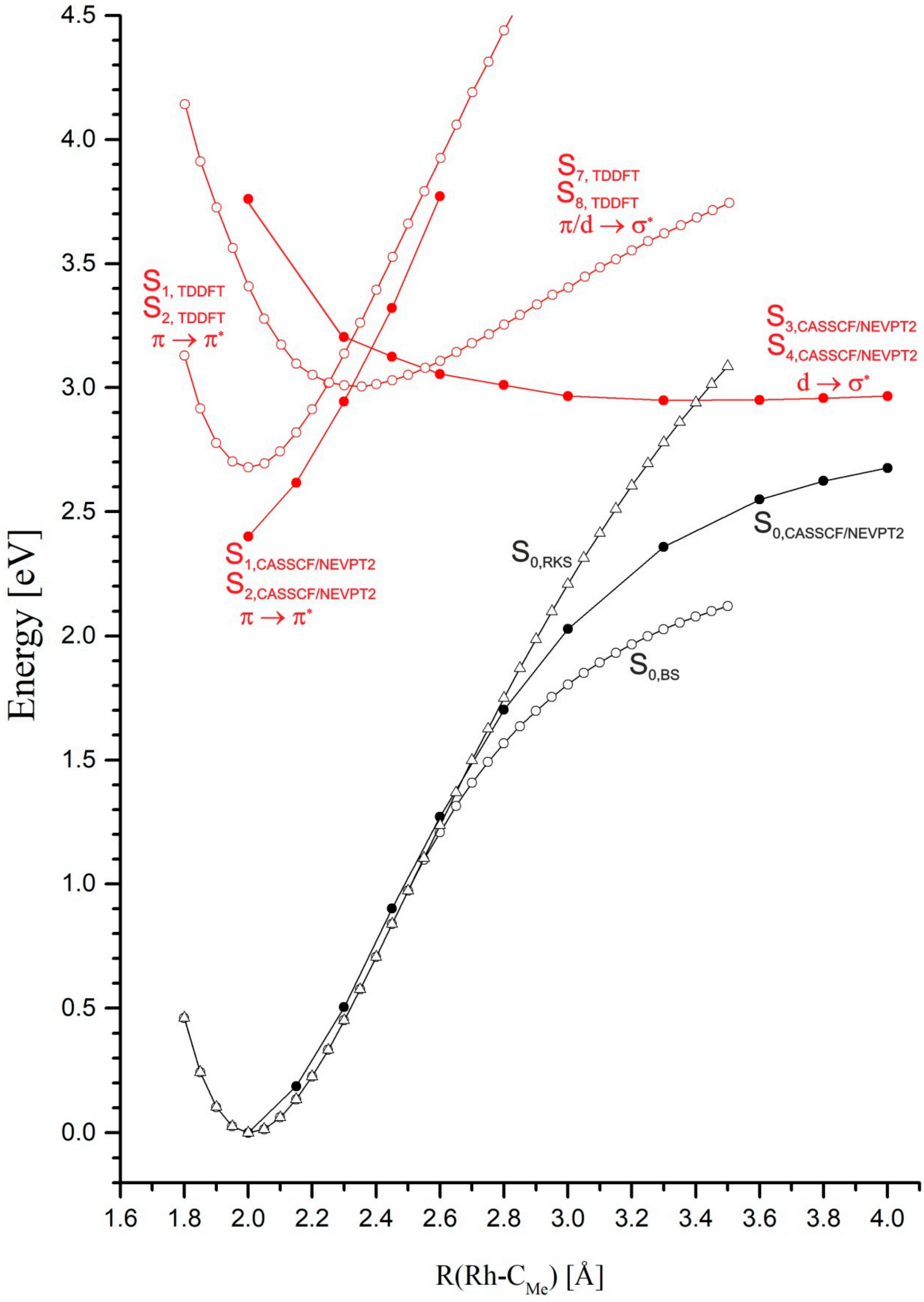

Figure S7. Comparison of the potential energy curves of the ground state S_0 and the lowest excited singlet state, obtained on the basis of calculations at the DFT, TDDFT, and CASSCF/NEVPT2 level of theory. Black lines - S_0 ground-state PECs; red lines - PECs of the lowest singlet excited state. Empty triangle and empty circles - PECs obtained using the DFT and TDDFT methods; filled circles - PECs determined based on CASSCF/NEVPT2 calculations. CASSCF/NEVPT2 calculations were performed for the optimised geometry of the MeRhPor complex at the level of the DFT/PBE0 method and for selected, frozen Rh-C_{Me} distances ranging from 2.00 Å to 4.00 Å. CASSCF calculations used an active CAS(12,12) space containing 12 electrons and 12 orbitals. The composition of the active space included rhodium orbitals $3d_{x2-y2}$, $3d_{xz}$, $3d_{yz}$, $3d_{xy}$, $4d_{xz}$, and

$4d_{yz}$, two occupied π and two unoccupied π* orbitals localised on the porphyrin ring and two σ and σ* orbitals describing the rhodium–carbon bond. CASSCF calculations were performed in the multistate option for 20 singlet states. The def2-TZVP basis function was applied for all atoms in the complex, and resolution of identity (RI) approximation was used. Calculations were performed using the continuous solvent model CPCM with benzene as a solvent. The multireference NEVPT2 method was applied in order to include dynamic correlation. References for CASSCF/NEVPT2 method: Roos, B. O. The Complete Active Space Self-Consistent Field Method and its Applications in Electronic Structure Calculations. In *Advances in Chemical Physics: Ab Initio Methods in Quantum Chemistry Part 2,* K. P. Lawley, K. P., Eds.; John Wiley & Sons, Ltd, 1987, Volume 69 pp. 399-445; Angeli, C.; Cimiraglia, R.; Evangelisti, S.; Leininger, T.; Malrieu, J.-P. Introduction of n-electron valence states for multireference perturbation theory. *J. Chem. Phys.* **2001**, *114*, 10252–10264; Angeli, C.; Cimiraglia, R.; Malrieu, J.-P. N-electron valence state perturbation theory: a fast implementation of the strongly contracted variant. *Chem. Phys. Lett.* **2001**, *350*, 297-305. Angeli, C.; Cimiraglia, R.; Malrieu, J.-P. n-electron valence state perturbation theory: A spinless formulation and an efficient implementation of the strongly contracted and of the partially contracted variants. *J. Chem. Phys.* 2002, *117*, 9138–9153.

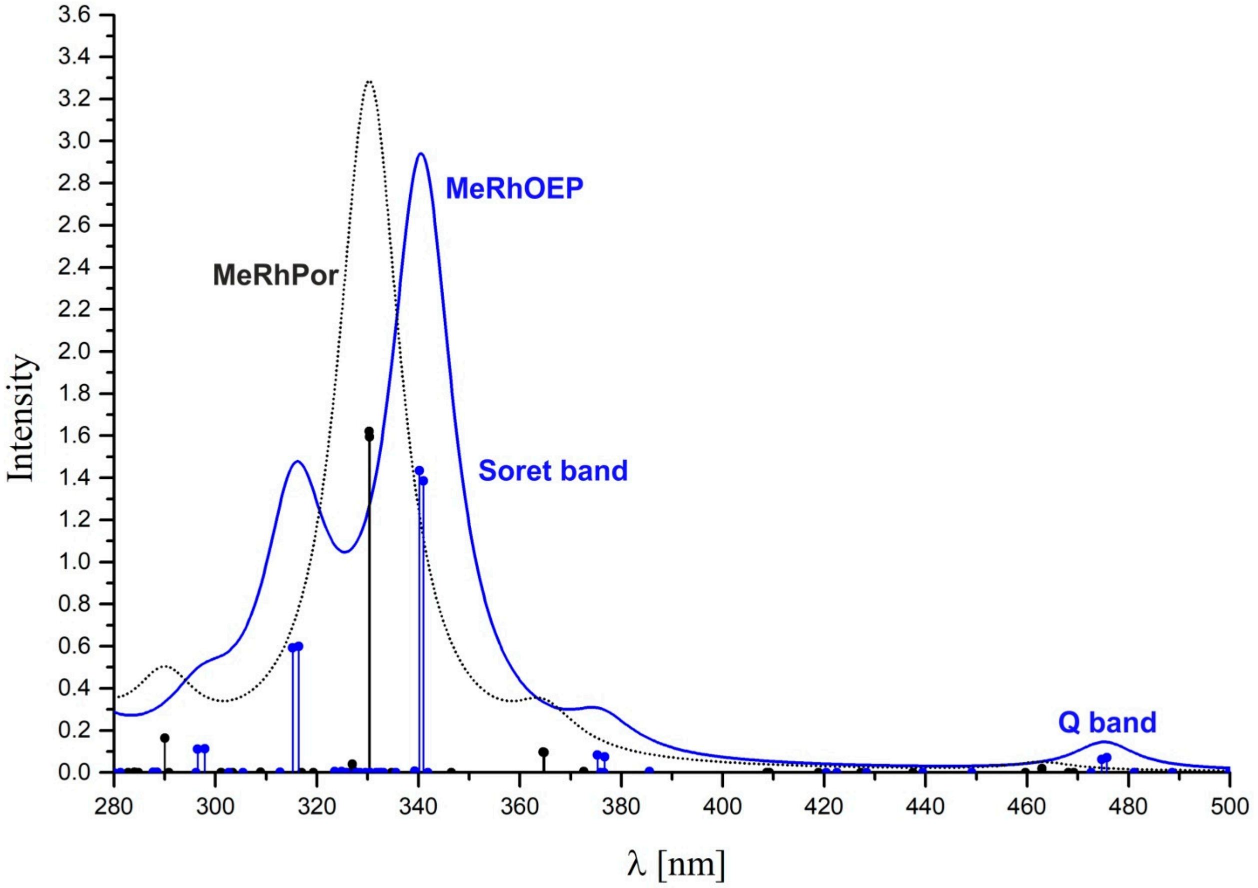

Figure S8. Simulated UV/VIS spectra for the MeRhOEP complex (blue solid line) and MeRhPor complex (black dashed line) obtained on the basis of the TDDFT calculations. The simulated spectral lines were obtained using Lorentzian broadening with a half-width of 15 nm. Vertical blue and black lines are calculated wavelengths for vertical excitations to singlet states for the MeRhOEP (blue line) and MeRhPor (black line) complexes, respectively. The height of the line corresponds to the calculated value of the oscillator strength f for individual electronic transitions.
